# The Effect of Molecular Structure on the Properties of Fluorene Derivatives for OLED Applications

**DOI:** 10.3390/molecules29204918

**Published:** 2024-10-17

**Authors:** Anna Pidluzhna, Aivars Vembris, Raitis Grzibovskis, Margarita Anna Zommere, Oleksandr Bezvikonnyi, Jurate Simokaitiene, Melita Baronaite, Dmytro Volyniuk, Juozas V. Grazulevicius, Amjad Ali, Glib Baryshnikov, Khrystyna Ivaniuk, Hryhorii Starykov, Pavlo Stakhira

**Affiliations:** 1 Institute of Solid State Physics, University of Latvia, Kengaraga Str. 8, LV-1063 Riga, Latvia; 2 Department of Polymer Chemistry and Technology, Faculty of Chemical Technology, Kaunas University of Technology, K. Baršausko St. 59, LT-51423 Kaunas, Lithuania; 3 Department of Physics, Faculty of Mathematics and Natural Sciences, Kaunas University of Technology, Studentų St. 50, LT-51369 Kaunas, Lithuania; 4 KTU “M-Lab” Laboratory Center, Kaunas University of Technology, Studentų St. 63A, LT-51369 Kaunas, Lithuania; 5 Laboratory of Organic Electronics, Department of Science and Technology, Linköping University, SE-60174 Norrköping, Sweden; 6 Department of Chemistry and Nanomaterials Science, Bohdan Khmelnytsky National University, 18031 Cherkasy, Ukraine; 7 Department of Electronic Engineering, Institute of Telecommunications, Radioelectronics and Electronic Engineering, Lviv Polytechnic National University, Stepan Bandera 12, 79013 Lviv, Ukraine

**Keywords:** fluorene derivatives, blue-emission, OLEDs, exciplex, electroplex

## Abstract

A new family of symmetrical fluorene derivatives with different types of substituents attached to the C-2 and C-7 positions of the fluorene core synthesized by the Sonogashira coupling reactions is reported. The electronic structures and the properties of the compounds investigated by means of photoelectron emission spectroscopy, UV–Vis absorption and photoluminescent spectroscopy as well as by DFT and TD-DFT theoretical calculations are discussed. It is shown that the nature of substituents influences the π-conjugation of the molecules. No intermolecular charge transfer within the investigated wavelength range is observed. The applicability of the synthesized compounds in organic light-emitting diodes (OLEDs) based on exciplex emission is demonstrated. The advanced co-deposition technique with the tuned OLED architecture was applied and resulted in improved OLED parameters.

## 1. Introduction

The global market of organic light-emitting diodes (OLEDs) is constantly growing. According to a recent forecast, it is expected to grow further by at least 13.6% annually for the next 5 years [1]. Such a forecast is caused by different reasons. First of all, the ecological situation in both global and local contexts is worsening [2]. This means that the world requires transformation towards novel technologies with low or zero greenhouse gas emissions to meet its carbon-neutral net zero targets [3,4]. OLEDs are promising devices because of their more eco-friendly and less energy-consuming technology of fabrication with respect to that of their inorganic counterparts, small weight, low-cost synthesis of materials, more energy-efficient performance and wide variety of design solutions [5].

A great deal of different types of emitters and hosts have been synthesized and investigated since the first thin-film electroluminescence was discovered in anthracene single crystal in 1963 [6]. Among organic π-conjugated substances with large energy band gaps, fluorene derivatives are considered promising emitters because of their good thermal stability, charge transport properties and high photoluminescence efficiency [7,8]. Fluorene derivatives can exhibit strong intermolecular interactions, such as aggregation-induced emission or excimer formation [9,10]. These interactions can lead to reduced device efficiency and undesirable emission characteristics [11]. Fluorene derivatives, particularly those with electron-donating or electron-accepting groups, can be sensitive to degradation, leading to a decrease in device lifetime and performance [12].

Many attempts have been made to tune the physical and optical properties of organic emitters towards better light-emissive performance by modification of the structure of molecules by using different building blocks for increasing π-conjugation length and improving the solubility and processability of the compounds [13,14,15]. However, ethynyl linkage has been relatively seldom used in the design of organic emitters so far.

The aim of this work was a synthesis of symmetrical derivatives of fluorene with the different substituents attached via ethynyl linkages and the investigation of their photophysical properties and applicability in organic optoelectronics. A series of OLEDs were fabricated by the thermal vacuum evaporation of the organic layers incorporating the various exciplex-emitting systems. The exciplex formation on the interfaces of layers and within the guest–host systems was achieved. The molecular features of the compounds that led to the improvement of exciplex-emission efficiency are discussed.

## 2. Results and Discussion

**Scheme 1:**
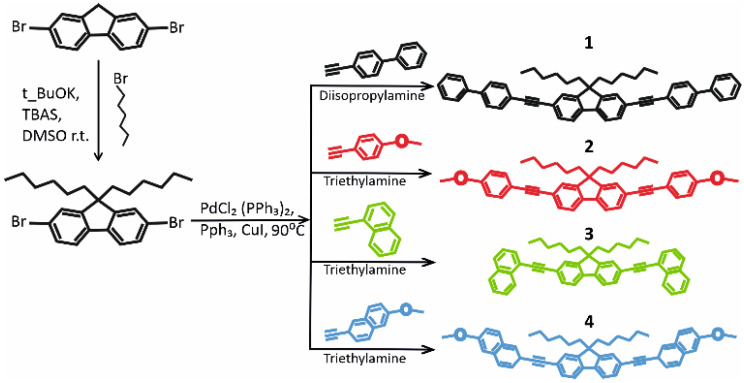
Synthesis of fluorene-based emitters **1**–**4**.

Synthesis. The fluorene derivatives were synthesized by the route shown in Scheme 1 [16]. The commercially available 2,7-dibromofluorene was treated with 1-bromohexane using t-BuOK to obtain 2,7-dibromo-9,9′-dihexylfluorene. Then, Sonogashira couplings of 2,7-dibromo-9,9-dihexylfluorene with the different phenylacethylene derivatives in the presence of PdCl2(PPh3)2, PPh3, CuI and diisopropylamine or triethylamine gave four derivatives of fluorene, i.e., 9,9-dihexyl-2,7-bis[2-(biphenyl-4-yl)ethynyl]fluorene (**1**), 9,9-dihexyl-2,7-bis[2-(4-methoxyphenyl)ethynyl]fluorene (**2**), 9,9-dihexyl-2,7-bis[2-(-(naphthalen-1-yl)ethynyl]fluorene (**3**), 9,9-dihexyl-2,7-bis[2-(6-methoxynaphthalen-2-yl)ethynyl]fluorene (**4**). Detailed information on the synthesis and the identification data including the NMR and IR data of compounds **1**–**4** can be found in the Supplementary Materials. This confirms the structures of the synthesized compounds.

Physical properties. Estimation of the energy gaps of the compounds was performed by photoconductivity measurement. Photoemission yield spectroscopy was used to obtain the ionization potential value for investigated materials. The method is used as the most suitable since the emitters are intended to be used for the formation of the layers of OLEDs by the thermal vacuum deposition technique. The data obtained are presented in Table 1.

After the attachment of electron-rich units the highest Occupied molecular orbital (HOMO) level usually rises up while the introduction of electron-deficient units results in the decrease of the lowest unoccupied molecular orbital (LUMO) energy [17,18,19,20]. This tendency is observable in the case of the synthesized materials. Compounds **2** and **4** containing methoxy groups demonstrate a higher HOMO energy level than compounds **1** and **3** (Table 1).

**Figure 1:**
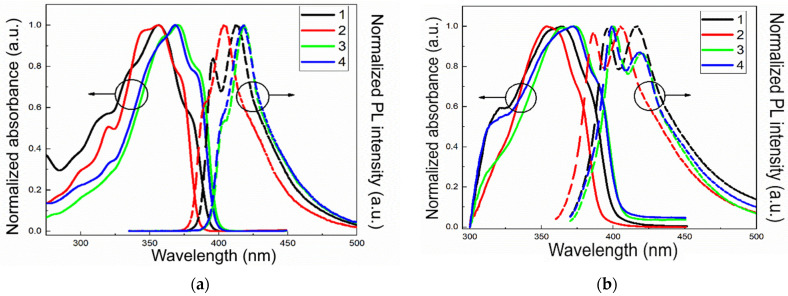
UV–Vis absorption spectra (straight lines) and photoluminescence spectra (dashed lines) with λ_ex_ = 360 nm for THF dilute solutions (10^−4^ M) of synthesized substances (**a**). UV–Vis absorption spectra (straight lines) and photoluminescence spectra (dashed lines) with λ_ex_ = 360 nm for encapsulated solid films of synthesized substances (**b**).

The normalized absorption spectra of THF solutions and of the solid films spin-coated on quartz substrates of compounds **1**–**4** are presented in Figure 1 and the main optical data are summarized in Table 1. The extinction coefficients for 10^−4^ M solutions at absorption maxima stayed in the range of 40,000–80,000 M^−1^cm^−1^.

The absorption spectra of THF solutions and thin films of the studied compounds show relatively wide peaks of typical profile for fluorene derivatives [21,22,23]. This peak is due to π–π* transition fluorene groups [19,24]. The extensions of π-conjugation bathochromically shift the position of the absorption peak from 355 nm for **2** to 370 nm for **4**. Compound **2** has the shortest conjugated system in the series of compounds. There are two benzene rings in compound **1** but they are linked via the C–C σ-bridge. Consequently, the π-conjugation between the rings is less efficient than that in **3** and **4**.

The absorption spectra of the solutions and films of compounds **3** and **4** are very similar with absorption maxima at around 370 nm and the almost identical position of the subpeaks. This observation shows negligible effect of the methoxy group on the absorption bands.

The emission spectra of the solutions of compounds **1**–**4** in THF are similar to those of fluorene derivatives [17]. The position of the emission maxima is in the same order as for the absorption maxima and is determined by π-conjugation. The emission spectra of the films of the synthesized compounds exhibit two sharp peaks at ca. 400 nm and 420 nm (Figure 1b) pointing to the vibrational fine structure. The wavelengths of the subpeaks are mostly the same for THF solutions and films of the compounds. It indicates a negligible change in dipole–dipole interactions when the compounds are dissolved in a polar solvent. Consequently, the photoluminescence of the compounds is characterized by the emission of local excited states. The emission spectra of the films undergo changes after continuous photoexcitation, specifically in the green range, due to photodegradation (Figure S1). Therefore, all experiments presented and discussed in the current work were performed with special attention paid to handling procedures and only measurements of samples encapsulated with quartz glass and epoxy resin were executed to avoid any additional influence.

Photoluminescence quantum yield measurements revealed values from 49 to 66% for THF solutions and from 53 to 59% for the solid films of the compounds (Table 1). Both the solutions and the films of compound **1** showed the lowest emission quantum yields. Despite the π-conjugation length in a molecule, the architecture of compound **1** resulted in the deepest-lying HOMO energy level affecting the conjugation and consequently lowering emission quantum yield of the corresponding radiative transitions from the singlet excited states in comparison with those of compounds **2**–**4**.

Multiexponential fit was carried out for the photoluminescence decay curves of the films of the compounds. The films exhibited lifetimes of up to 2.6 ns indicating prompt fluorescence of the local excited states in correspondence with the previously reported fluorene derivatives [17,24] (Table S1). Two components of the PL decay curves correspond to the different emissive transitions manifested by two emission subpeaks.

**Figure 2:**
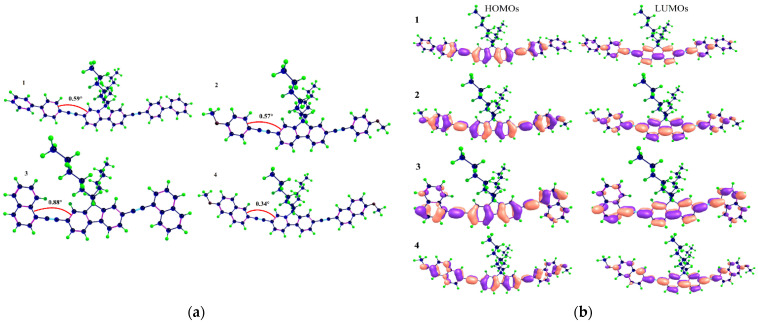
Optimized geometries of chemical structures of **1**–**4** (**a**). HOMO and LUMO orbitals of the molecules (iso value 0.02) in THF solution (**b**).

Theoretical Calculations. To verify our experimental results theoretical calculations were performed. Figure 2 depicts the optimized geometries and frontier molecular orbital maps of all the designed structures.

**Figure 3:**
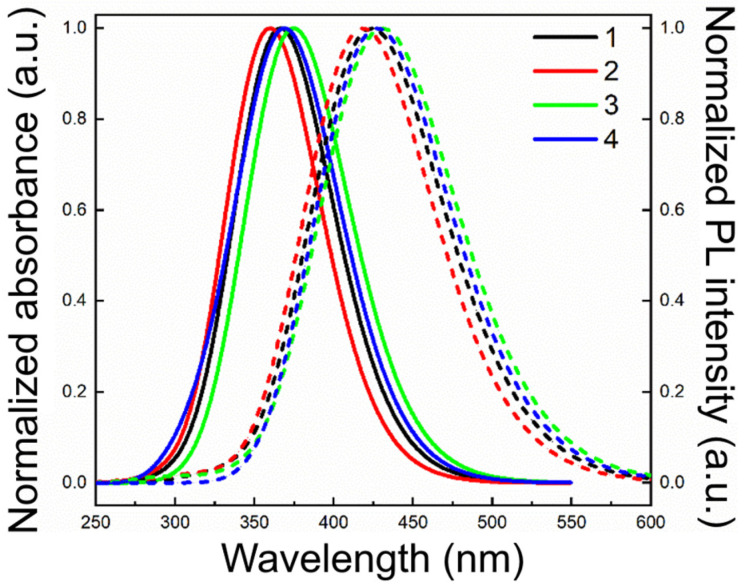
Absorption (solid lines) and fluorescence (dashed lines) spectra of all dyes computed with TD-DFT-CAM-B3LYP level of theory and 6-31G(d) basis set.

Figure 3 represents the absorption and fluorescence spectra of all dyes computed with TD-DFT-CAM-B3LYP level of theory and 6-31G(d) basis set (Gaussian 16) [25]. The detailed description of the computational methodology is in SI. Table 2 lists the HOMO, LUMO energy levels and HOMO–LUMO gap. Table 3 compares the experimental and theoretical data regarding absorption and fluorescence.

The HOMOs and LUMOs for molecules **1**–**4** are localized on the fluorene core and end units showing the HOMO–LUMO overlap, due to the presence of carbon–carbon triple bonds, which increase the conjugation between the central and end moieties (Figure 2). These maps demonstrate that the excitations are not charge-transfer transitions. The higher HOMO–LUMO gap of **1** when compared with **3** and **4** is probably due to the carbon–carbon single bond between the two benzene rings in the end moieties that decreases the π-conjugation between them. Moreover, the dihedral angles between fluorene cores and end groups range from 0.34° to 0.88°, with the lowest value for **4** and the highest one for **3**.

These small angles demonstrate the planarity of these molecules which in turn increase the conjugation between central and end fragments. The lower torsion angles between fluorene and end units due to the C–C triple bond result in higher HOMO–LUMO orbitals overlap and can be seen very clearly in Figure 2. The HOMO–LUMO gaps obtained from DFT calculations follow the order **3** < **4** < **1** < **2** with the values of 3.27 < 3.28 < 3.36 < 3.43 eV. Normalized fluorescence and UV–Vis spectra of all dyes are illustrated in Figure 3 and the comparison between experimental and theoretical data is given in Table 3. The computed absorption and emission maxima are in good agreement with the experimental ones following the order **2** < **1** < **4** ≤ **3**.

The values of absorption maxima of **1**, **2**, **3** and **4** are 366, 360, 375 and 370 nm, respectively (Figure 3). The corresponding values for fluorescence are 424, 418, 430 and 427 nm. Similar to the experimental results, the theoretical absorption and emission spectra for **1–4** are typical for fluorene derivatives and correspond to the π–π* transitions of conjugated molecules. The theoretical Stokes shifts in THF solvent also reveal the stable planar configuration (Table 3). Introducing different end units on both sides of the fluorene alters the photophysical properties of the symmetrical molecules. The absorption and emission spectra of **3** and **4** are theoretically redshifted when compared with **1** and **2** demonstrating the crucial role of conjugated end units and their effect on the photophysical properties of the molecules.

**Figure 4:**
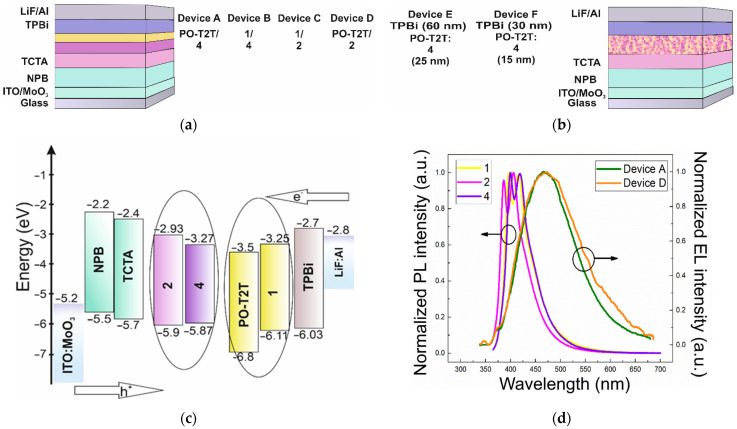
The structure of the OLED devices A–D (**a**) and E, F (**b**). Equilibrium energy level diagram (**c**) The EL spectra for device A and D vs. the solid film PL spectra of substances **1**, **2** and **4** (**d**).

Characterization of OLED. Exciplexes based on the presented emitter were considered for the investigation of electroluminescence (EL) aiming at boosting the intermolecular charge transfer. Therefore, the structure for exciplex-emissive OLEDs was developed as follows: ITO/MoO_3_/NPB/TCTA/exciplex system/TPBi/LiF/Al. The full description is in SI. In the devices, the MoO_3_ was used for hole injecting and LiF was used for electron injecting. NPB (*N*,*N*′-Di(1-naphthyl)-*N*,*N*′-diphenyl-(1,1′-biphenyl)-4,4′-diamine) and TPBi (2,2′,2′′-(1,3,5-Benzinetriyl)-tris(1-phenyl-1-*H*-benzimidazole)) were selected for deposition of hole and electron transport layers, respectively. TCTA (4,4′,4′′-tris(carbazol-9-yl)triphenylamine) was employed for hole transport and electron/exciton blocking. Exciplex-forming systems were chosen as bilayers of **4**/PO-T2T, **4**/**1**, **2**/**1**, **2**/PO-T2T for OLEDs A-D, respectively. Figure 4a–c represents the detailed structure of fabricated organic light-emitting devices and a schematic energy diagram for described OLEDs. As the structure for OLEDs was developed in a way to obtain the interface exciplex emission, compounds **2** and **4** were used as donors and **1** and PO-T2T were selected as acceptors. The parameters of prepared devices are listed in Table S2.

**Figure 5:**
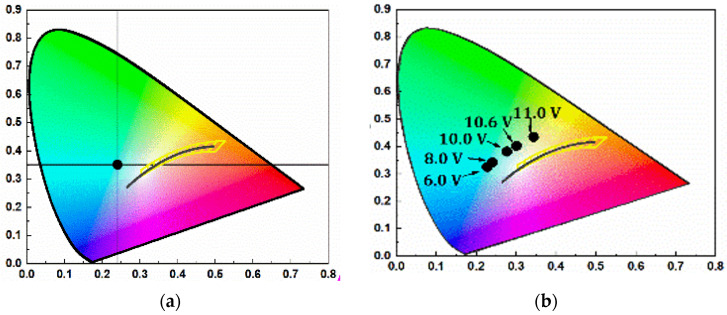
1931 CIE coordinates of OLED A (**a**) and D (**b**) at different voltages.

The fabricated devices emit the overall white–blue color which is confirmed by the EL spectra (see Figure 4d and Figures S3–S6) and the 1931 CIE chromaticity diagram proving the exciplex formation (Figure 5) [26]. The EL spectra containing the bands assigned to the exciplex emission totally correspond to the PL spectra of the respective films (Figure S2). The efficiency of the investigated devices is relatively low for all samples apart from device A (**4**/PO-T2T) which had the highest brightness among them, which can be explained by the lowest value for the dihedral angle between the fluorene core and end groups for sample **4** as was shown by the theoretical calculations. The devices based on **4** are also characterized by lower turn-on voltages than OLEDs with the emitting layers based on the other exciplex-emitting systems (Table S2, Figures S7 and S8). This observation proves the role of effective charge balance in the improvement of the efficiency of the devices [27].

The lowest dihedral angle assures better planarity which in turn enables better exciplex formation between the layers of **4** and the acceptor compounds. This fact is reflected by the brightness values listed in Table S2.

In the case of the EL spectra of OLEDs, other peaks than the one assigned to the exciplex band with the maximum at 405 nm are also observable which confirm the partial presence of emission related to the π–π* transition located on a fluorene moiety. The EL spectra of OLED D (Figure S4) show the expressive shift in emission maxima from 505 nm at 6 V of applied voltage and gradually to 555 nm at 11 V. At the same time, the shoulder in a red region becomes more and more prominent upon increasing voltage. This phenomenon can be explained by the formation of electroplexes [26] with the participation of side groups of substance **2** when emission takes place mainly from the direct irradiative recombination of holes and electrons residing at two neighboring molecules. Electroplexes are formed due to increased electrical excitation which is also responsible for the saturation of triplet population. Thus, upon increasing voltage the phenomena of triplet–triplet annihilation and triplet–polaron quenching cause the efficiency roll-off [28]. The spectral shifts observed at the different voltages are related to the electroplexes and not to the triplet-facilitated phenomena responsible for the efficiency roll-off. The structure of compound **4** is more planar when compared to **2**, and the higher dihedral angle in **2** assures tension in the alignment of film interfaces of **2** donor and PO-T2T acceptor, so that the formation of excited states between them became dependent on the applied voltage. The increase in applied voltage enables electromer formation and the emission maxima shift to the red region as a result.

The OLED structure was further optimized. The thickness of layers was reduced to improve the charge balance on recombination sites [27]. Additionally, the emitting layers of different thicknesses were composed of donor and acceptor compounds **4** and PO-T2T mixed together and deposited by the wet coating technique (Table S2, Devices E and F). The combination of wet coating and thermal vacuum evaporation together with doping of the emitting layer resulted in the increase of external quantum efficiency from 0.18 to 0.44% and the blue shift of the EL spectra.

## 3. Materials and Methods

2,7-dibromofluorene, 1-bromohexane, t-BuOK, TBAS, DMSO, 4-ethynyl-1,1′-biphenyl, triphenylphosphine, diisopropylamine, CuI, PdCl_2_(PPh_3_)_2_, 1-ethynyl-4-methoxybenzene triethylamine, 1-ethynylnaphthalene, 2-ethynyl-6-methoxynaphthalene, and sodium sulfate were purchased from Sigma Aldrich or Fluorochem and used as received. 2,7-Dibromo-9,9-dihexylfluorene was prepared according to the published procedure.


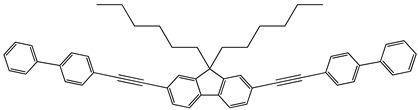

**9,9-Dihexyl-2,7-bis[2-(biphenyl-4-yl)ethynyl]fluorene (1)**

2,7-Dibromo-9,9-dihexylfluorene (1.26 g, 2.55 mmol) and 4-ethynylbiphenyl (1 g, 5.61 mmol) were dissolved in di-isopropylamine (15 mL), and the solution was degassed. Then, CuI (0.0043 g, 0.022 mmol), PdCl_2_(PPh_3_)_2_ (0.0311 g, 0.044 mmol), and triphenylphosphine (0.0348 g, 0.133 mmol) were added to the reaction mixture under inert atmosphere, and it was stirred at 90 °C for 20 h. The crude product was extracted with DCM/water, and the organic layer was washed with saturated brine solution, dried over Na_2_SO_4_, and concentrated under reduced pressure. The product was purified by column chromatography on silica gel using hexane as the eluent. The product was recrystallized from hexane (mp: 182 °C). The yield of the product was 34% (0.59 g).

^1^H NMR (400 MHz, CDCl_3_), δ (m. d.): 0.58–0.67 (m, 4H, –CH_2_–), 0.78 (t, 6H, –CH_3_, *J* = 7.1 Hz), 1.00–1.16 (m, 12H, –CH_2_–), 1.97–2.03 (m, 4H, –CH_2_–), 7.34–7.39 (m, 2H, Ar), 7.46 (t, 4H, Ar, *J* = 7.6 Hz), 7.54 (s, 2H, Ar), 7.55–7.57 (m, 1H, Ar), 7.58 (s, 1H, Ar), 7.60 (s, 2H, Ar), 7.63 (d, 9H, Ar, *J* = 7.5 Hz), 7.65–7.69 (m, 3H, Ar).

^13^C NMR (100 MHz, CDCl_3_), δ (m.d.): 14.09; 22.70; 23.80; 29.80; 31.62; 40.50; 55.31; 89.74; 91.20; 120.04; 121.98; 122.28; 125.99; 127.06; 127.10; 127.17; 127.70; 127.91; 128.92; 128.96; 130.83; 132.05; 133.00; 140.36; 140.75; 140.94; 151.17.

IR, *n*, (cm^−1^): 3031 (Ar C–H); 2925, 2856 (Alif. C–H); 1598, 1580, 1517, 1482 (Ar C=C); 1463, 1447, 1379 (Alif. C–H); 1006, 896, 839, 826, 761 (Ar C–H); 722 (Alif. C–H); 695 (≡C–H).

MS (ACPI+, 25 V), *m*/*z* (%): 687 ([M + H]^+^, 100).


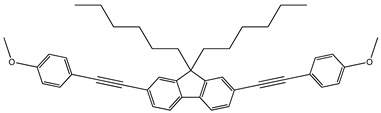

**9,9-Dihexyl-2,7-bis[2-(4-methoxyphenyl)ethynyl]fluorene (2)**

2,7-Dibromo-9,9-dihexylfluorene (1.69 g, 3.44 mmol) and 1-ethynyl-4-methoxybenzene (1 g, 7.57 mmol) were dissolved in triethylamine (15 mL), and the solution was degassed. Then, CuI (0.0058 g, 0.030 mmol), PdCl_2_(PPh_3_)_2_ (0.0420 g, 0.060 mmol) and triphenylphosphine (0.0469 g, 0.179 mmol) were added to the reaction mixture under inert atmosphere and it was stirred at 90 °C for 20 h. The crude product was extracted with DCM/water, the organic layer was washed with saturated brine solution, dried over Na_2_SO_4_ and concentrated under reduced pressure. The product was purified by column chromatography on silica gel using hexane as the eluent. The product was recrystallized from hexane (mp: 143 °C). The yield of the product was of 72% (1.48 g).

^1^H NMR (400 MHz, CDCl_3_), δ (m. d.): 0.56–0.65 (m, 4H, –CH_2_–), 0.77 (t, 6H, –CH_3_, *J* = 7.1 Hz), 0.99–1.16 (m, 12H, –CH_2_–), 1.94–2.01 (m, 4H, –CH_2_–), 3.83 (s, 6H, –OCH_3_), 6.87–6.92 (m, 4H, Ar), 7.47–7.53 (m, 8H, Ar), 7.64 (dd, 2H, Ar, *J* = 0.9 Hz, *J* = 7.6 Hz).

^13^C NMR (100 MHz, CDCl_3_), δ (m.d.): 13.99; 22.62; 23.72; 29.73; 31.55; 40.45; 55.18; 55.28; 89.13; 89.67; 114.03; 114.11; 115.45; 119.84; 122.15; 125.75; 130.54; 133.01; 134.02; 140.43; 151.01; 159.59.

IR, *n*, (cm^−1^): 3060, 3010 (Ar C–H); 2926, 2841 (Alif. C–H); 1601, 1568, 1509 (Ar C=C); 1463, 1439, 1377 (Alif. C–H); 1287, 1245, 1172, 1108, 1023 (C–O–C); 896, 829, 811 (Ar C–H); 721 (Alif. C–H); 657 (≡C–H).

MS (ACPI+, 25 V), *m*/*z* (%): 596 ([M + H]^+^, 100).


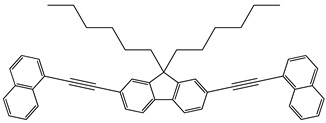

**9,9-Dihexyl-2,7-bis[2-(naphthalen-1-yl)ethynyl]fluorene (3)**

2,7-Dibromo-9,9-dihexylfluorene (1.47 g, 2.99 mmol) and 1-ethynylnaphthalene (1 g, 6.57 mmol) were dissolved in triethylamine (15 mL) and the solution was degassed. Then CuI (0.0050 g, 0.026 mmol), PdCl_2_(PPh_3_)_2_ (0.0365 g, 0.052 mmol) and triphenylphosphine (0.0407 g, 0.155 mmol) were added to the reaction mixture under inert atmosphere and it was stirred at 90 °C for 20 h. The crude product was extracted with DCM/water, the organic layer was washed with saturated brine solution, dried over Na_2_SO_4_ and concentrated under reduced pressure. The product was purified by column chromatography on silica gel using hexane as the eluent. The product was recrystallized from hexane (mp: 161 °C). The yield of the product was of 72% (1.37 g).

^1^H NMR (400 MHz, CDCl_3_), δ (m. d.): 0.62–0.72 (m, 4H, –CH_2_–), 0.77 (t, 6H, –CH_3_, *J* = 7.0 Hz), 1.02–1.17 (m, 12H, –CH_2_–), 2.02–2.07 (m, 4H, –CH_2_–), 7.48 (dd, 2H, Ar, *J* = 7.2 Hz, *J* = 8.2 Hz,), 7.52–7.57 (m, 2H, Ar), 7.61–7.68 (m, 6H, Ar), 7.72 (s, 1H, Ar), 7.74 (s, 1H, Ar), 7.81 (dd, 2H, Ar, *J* = 1.1 Hz, *J* = 7.2 Hz), 7.85 (d, 2H, Ar, *J* = 8.3 Hz), 7.88 (d, 2H, Ar, *J* = 8.1 Hz), 8.52 (d, 2H, Ar, *J* = 8.4 Hz).

^13^C NMR (100 MHz, CDCl_3_), δ (m.d.): 14.01; 22.62; 23.76; 29.72; 31.55; 40.42; 55.36; 87.93; 95.38; 120.05; 121.01; 122.12; 125.32; 125.94; 126.27; 126.45; 126.77; 128.34; 128.72; 130.38; 130.90; 133.25; 140.81; 151.23.

IR, *n*, (cm^−1^): 3057 (Ar C–H); 2951, 2924, 2854 (Alif. C–H); 1584, 1506 (Ar C=C); 1468, 1395, 1374 (Alif. C–H); 889, 823, 797, 770 (Ar C–H); 723 (Alif. C–H); 673 (≡C–H).

MS (ACPI+, 25 V), *m*/*z* (%): 635 ([M + H]^+^, 100).


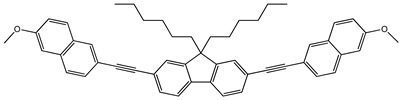

**9,9-Dihexyl-2,7-bis[2-(6-methoxynaphthalen-2-yl)ethynyl]fluorene (4)**

2,7-Dibromo-9,9-dihexylfluorene (1.23 g, 2.49 mmol) and 1-ethynylnaphthalene (1 g, 5.49 mmol) were dissolved in triethylamine (15 mL) and the solution ws degassed. Then CuI (0.0042 g, 0.022 mmol), PdCl_2_(PPh_3_)_2_ (0.0305 g, 0.043 mmol) and triphenylphosphine (0.0340 g, 0.130 mmol) were added to the reaction mixture under inert atmosphere and stirred at 90 °C for 20 h. The crude product was extracted with DCM/water, the organic layer was washed with saturated brine solution, dried over Na_2_SO_4_ and concentrated under reduced pressure. The product was purified by column chromatography on silica gel using hexane as eluent. The product was recrystallized from hexane (mp: 182 °C). The yield of the product was of 54% (0.94 g).

^1^H NMR (400 MHz, CDCl_3_), δ (m. d.): 0.59–0.68 (m, 4H, –CH_2_–), 0.77 (t, 6H, –CH_3_, *J* = 7.1 Hz), 1.00–1.16 (m, 12H, –CH_2_–), 1.97–2.03 (m, 4H, –CH_2_–), 3.91 (s, 6H, –OCH_3_), 7.12 (d, 2H, Ar, *J* = 2.4 Hz), 7.17 (dd, 2H, Ar, *J* = 2.5 Hz, *J* = 8.9 Hz), 7.54–7.57 (m, 4H, Ar), 7.59 (dd, 2H, Ar, *J* = 1.6 Hz, *J* = 8.5 Hz), 7.66 (s, 1H, Ar), 7.68 (s, 1H, Ar), 7.70 (d, 2H, Ar, *J* = 4.6 Hz), 7.72 (d, 2H, Ar, *J* = 5.1 Hz), 8.02 (s, 2H, Ar).

^13^C NMR (100 MHz, CDCl_3_), δ (m.d.): 14.00; 22.63; 23.76; 29.74; 31.56; 40.46; 55.23; 55.32; 90.16; 90.36; 105.84; 118.23; 119.41; 119.93; 122.08; 125.91; 126.85; 128.54; 128.99; 129.32; 130.70; 131.22; 134.10; 140.60; 151.09; 158.32.

IR, *n*, (cm^−1^): 3061 (Ar C–H); 2926, 2856 (Alif. C–H); 1627, 1599, 1499, 1484 (Ar C=C); 1462, 1390 (Alif. C–H); 1261, 1209, 1163, 1126, 1029 (C–O–C); 886, 855, 848, 819, 803, 754 (Ar C–H); 724 (Alif. C–H); 662 (≡C–H).

MS (ACPI+, 25 V), *m*/*z* (%): 696 ([M + H]^+^, 100).

*Thin film sample preparation and investigation*. The solutions of the compounds in THF with the concentration of 15 mg/mL were prepared. Then, the quartz substrates were washed and dried with nitrogen flow at room temperature. The thin films were fabricated by spin-coating technique in a static regime by placing 0.2 mL of the investigated solution onto the quartz substrate and spinning with a 800 rpm spin speed for 1min. Then, the samples were placed onto a hot-plate heated to 70 °C and kept there for 15 min. After cooling to room temperature (RT), the samples were encapsulated with Ossilla encapsulation resin and kept under UV light for 60 min to let the resin react and harden.

*Measurements*. The ionisation energy and electron affinity energy values of the compounds were determined from the photoelectron emission spectra and spectral photoconductivity measurements. The thin films for these experiments were prepared by spin-coting techniques on glass/ITO substrates. Solution concentration and spinning parameters were the same as those for the samples for photophysical measurements. Afterwards, the samples were dried at 70 °C for 15 min and deposited Al electrodes by thermal evaporation in a vacuum onto the films of the investigated compounds.

The photoemission yield spectroscopy and spectral photoconductivity measurements were conducted under vacuum conditions with a pressure of ca. 10^−5^ mBar. A custom-built measurement system was utilized for this purpose. An ENERGETIQ laser-driven light source (LDLS EQ-99, Wilmington, MA, USA) served as the UV radiation source. The spectral range of the measurements was from 4 to 6.5 eV. The incident photon energy was controlled using a diffraction grating monochromator (MYM-1, Spectral Products, Putnam, CT, USA) with a step of 0.05 eV. During the experiments, the samples were exposed to radiation through a quartz window in a vacuum chamber. The distance between the sample and the electrode for electron collection was ca. 2 cm. For electrical current measurements and voltage supply, a Keithley 617 electrometer (Cleveland, OH, USA) was employed.

Carry 7000 spectrtometer (Agilent, Santa Clara, CA, USA) was used to measure UV-Vis absorption spectra of THF solutions and the solid films of the synthesised compounds. An Edinburg Instruments Fluorescence Spectrometer FLS1000 (Livingston, UK) equipped with an integrating sphere was used for the investigation of emission properties and photoluminescence dynamics.

*Computational methodology*. The DFT and TD-DFT calculations were performed with Gaussian 16 software [25]. The optimized ground state geometries were found by a standard process of force-minimization. The vibrational frequencies were confirmed, and no imaginary frequency was found. The ground-state geometries’ optimization calculations were carried out using the B3LYP functional with a 6-31G(d) basis set. The same basis set with a CAM-B3LYP DFT functional was employed to perform the absorption and fluorescence spectra simulation. The DFT and TD-DFT calculations were performed for the THF solvent, the same approach was used in the experiment, and the dispersion corrections were considered. The Polarizable Continuum Model (PCM) is the model most commonly used to consider the solvent effects for excited states of organic compounds [29]. Therefore, the solvent effects were considered using the PCM approach. Accounting for the solution cavity is very crucial for real-time simulation and significant theory/experiment comparisons.

*OLED preparation and characterization*. Glass substrates covered with indium tin oxide (ITO) (15 Ω/square) were purchased from Präzisions Glas & Optik GmbH (Iserlohn, Germany). ITO substrates were cleaned according to the following procedure: (1) sonication in CHCl_3_; (2) sonication in acetone; (3) rinsing with deionized (DI) water; (4) sonication in 3 vol% of Hellmanex II solution; (4) rinsing with DI water; (5) sonication in DI water and isopropyl alcohol. After drying for the innitrogen flow, the samples were moved from the glovebox to the vacuum chamber in a sealed container for thermal evaporation of OLED layers.

Four devices were prepared by the described above method:

Device A: ITO/MoO_3_ (0.4 nm)/NPB (30 nm)/TCTA (15 nm)/4 (15 nm)/PO-T2T (10 nm)/TPBi (60 nm)/LiF (0.4 nm)/Al (60nm);

Device B: ITO/MoO_3_ (0.4 nm)/NPB (30 nm)/TCTA (15 nm)/4(15nm)/1 (10 nm)/TPBi (60 nm)/LiF (0.4 nm)/Al (60nm);

Device C: ITO/MoO_3_ (0.4 nm)/NPB (30 nm)/TCTA (15 nm)/2 (15 nm)/1 (10 nm)/TPBi (60 nm)/LiF (0.4 nm)/Al (60nm);

Device D: ITO/MoO_3_ (0.4 nm)/NPB (30 nm)/TCTA (15 nm)/2 (15 nm)/PO-T2T (10 nm)/TPBi (60 nm)/LiF (0.4 nm)/Al (60 nm).

The second set of OLEDs was made according to the solvent premixed deposition technique. The emissive layers were made from the 1 mL solution of 4 and PO-T2T (1:1 weight ratio) in degassed THF. The concentration of 4 and PO-T2T was 8.5 mg/mL for Device E and 5 mg/mL for Device F.

Device E: ITO/MoO_3_ (0.4 nm)/NPB (30 nm)/TCTA (15 nm)/4:PO-T2T (25 nm)/TPBi (60 nm)/LiF (0.4 nm)/Al (60 nm);

Device F: ITO/MoO_3_ (0.4 nm)/NPB (30 nm)/TCTA (15 nm)/4:PO-T2T (15 nm)/TPBi (30 nm)/LiF (0.4 nm)/Al (60 nm).

The current–voltage characteristics of as-prepared OLEDs were measured by the multimeter Keithley 2700. The electroluminescence brightness characteristics were measured by Konica Minolta Luminance and Color Meter CS-150 (Tokyo, Japan).

## 4. Conclusions

Four new symmetrical fluorene derivatives with different types of backbone substituents at the C-2 and C-7 positions of the fluorene core were synthesized by the Sonogashira coupling reaction. The synthesized compounds are photoactive because of the π–π* excitation which Occurs in fluorene moiety. Theoretical calculations confirmed the conclusions made based on photophysical data about the role of substituents on the planarity and the extension of π-conjugation. Thus, the lower torsion angles between fluorene and the end units due to the C–C triple bond led to the substantial overlap of the frontier molecular orbitals. The emitters possess deep energy levels of the highest Occupied molecular orbitals of ca. -6 eV. These energies are suitable for the formation of exciplexes in organic light-emitting diodes. The exciplexes are formed at the interfaces and within the emitting layers of donor material doped into an acceptor. It was shown that some of the devices operate through the excited states of electroplexes. The fabricated OLEDs emit in the blue–white region of the spectrum. Among four developed OLEDs, the device with the newly synthesized 9,9-dihexyl-2,7-bis[2-(6-methoxynaphthalen-2-yl)ethynyl]fluorene (4) as donor and commercially purchased PO-T2T used as acceptor exhibited the best performance.

## Figures and Tables

**Table 1:** Photophysical characteristics of compounds **1**–**4**.

Compound	Solutions				Solid Films							
	λ^ab^_max_, nm	λ^em^_max_, nm	Stokes shift, nm	PLQY, %	λ^ab^_max_, nm	λ^em^_max1_, nm	λ^em^_max2_, nm	Stokes shift, nm	PLQY, %	IP, eV	Eth, eV	EA, eV
**1**	356	396/412	40	49	364	396	416	32	53	6.11	2.87	3.24
**2**	355	404	49	61	353	386	406	33	58	5.90	2.97	2.93
**3**	371	418	47	63	368	400	419	32	59	6.01	2.70	3.31
**4**	369	418	49	66	371	399	419	28	57	5.88	2.61	3.27

λ^ab^_max_, nm is the position of absorption maximum in nm; λ^em^_max_, nm is the position of emission maximum in nm; PLQY, % photoluminescence quantum yield in %; IP, eV is Ionisation Potential in eV; Eth, eV is the threshold of intrinsic conductivity; EA, eV is electron affinity.

**Table 2:** The computed values of HOMO, LUMO energy levels and HOMO–LUMO gaps in THF solution.

Molecule	HOMO (eV)	LUMO (eV)	HOMO–LUMO Gap (eV)
**1**	−5.30	−1.94	3.36
**2**	−5.15	−1.72	3.43
**3**	−5.26	−1.99	3.27
**4**	−5.14	−1.86	3.28

**Table 3:** Comparison of the experimental maximum absorption and emission values with the theoretical values computed with TD-DFT-CAM-B3LYP level of theory and 6-31G(d) basis in THF solution.

Molecule	Experimental Data			Theoretical Data		
	λ^ab^_max_ (nm)	λ^em^_max_ (nm)	Stoke Shift (nm)	λ^ab^_max_theo_(nm)	λ^em^_max_theo_ (nm)	Stoke Shift (nm)
**1**	356	396/412	40	366	424	58
**2**	355	404	49	360	418	58
**3**	371	418	47	375	430	55
**4**	369	418	49	370	427	57

## Data Availability

The data presented in this study are available on request from the corresponding author.

## Supplementary Materials

The following supporting information can be downloaded at https://www.mdpi.com/article/10.3390/molecules29204918/s1: Table S1. Emission decay characteristics of the solid samples compounds **1**–**4**, Figure S1. Photoluminescence intensity of compounds **1**–**4** in thin-film state without encapsulation, Figure S2. Photoluminescence intensity of compounds **2** and **4** in thin-film state encapsulated (magenta and yellow plots) and in mixture with PO-T2T (orange and violet plots), Table S2. EL characteristics of the fabricated devices, Figure S3. The brightness and CIE1931 chromaticity diagram (a) and EL spectra of device A at the different applied voltages (b), Figure S4. The brightness and CIE1931 chromaticity diagram (a) and EL spectra of device D at the different applied voltages (b), Figure S5. The brightness and CIE1931 chromaticity diagram (a) and EL spectra of device E at the different applied voltages (b), Figure S6. The brightness and CIE1931 chromaticity diagram (a) and EL spectra of device F at the different applied voltages (b), Figure S7. Current density—voltage plots for devices A–D, Figure S8. Current density—voltage plots for devices A, E, F. Cartesian coordinates.

## References

[1] Xie G., Xue Q. (2023). Organic Light-Emitting Diodes. Encycl. Mater. Electron..

[2] Tollefson J. (2021). COVID curbed carbon emissions in 2020—But not by much. Nature.

[3] Jasiński J., Kozakiewicz M., Sołtysik M. (2021). The effectiveness of energy cooperatives operating on the capacity market. Energies.

[4] Kheirinejad S., Bozorg-Haddad O., Singh V.P., Loáiciga H.A. (2022). The effect of reducing per capita water and energy uses on renewable water resources in the water, food and energy nexus. Sci. Rep..

[5] Burroughes J.H., Bradley D.D.C., Brown A.R., Marks R.N., Mackay K., Friend R.H., Burns P.L., Holmes A.B. (1990). Light-emitting diodes based on conjugated polymers. Nature.

[6] Pope M., Kallmann H.P., Magnante P. (1963). Electroluminescence in organic crystals. J. Chem. Phys..

[7] Feng L.G., Hui L.J. (2017). Investigation on three-photon absorption induced upconversion fluorescence properties of two fluorene-based derivatives. Optik.

[8] Feng L.G., Wang T.H., Hui L.J. (2020). Experimental and quantum chemical studies of the structural enhancement of three-photon absorption in two symmetrical fluorene derivatives. Optik.

[9] Megha, Kumar V., Kaur P., Singh K. (2023). Julolidine based red emitting ESIPT/AIE active material showing luminescence beyond excimer emission: An “on-off” emission response to Cu^2+^. Spectrochim. Acta Part A Mol. Biomol. Spectrosc..

[10] Thirion D., Romain M., Rault-Berthelot J., Poriel C. (2012). Intramolecular excimer emission as a blue light source in fluorescent organic light emitting diodes: A promising molecular design. J. Mater. Chem..

[11] Gu J., Li Z., Li Q. (2023). From single molecule to molecular aggregation science. Coord. Chem. Rev..

[12] Yuan X., Tang W., Liu X., Jiang H. (2023). Synthesis and characterization of blue light emitters based on dimers of fluorene: Effects of different pendant electron-withdrawing moieties. Synth. Met..

[13] Ledwon P. (2019). Recent advances of donor-acceptor type carbazole-based molecules for light emitting applications. Org. Electron..

[14] Ledwon P., Pluczyk S., Idzik K.R., Beckert R., Lapkowski M. (2013). Bipolar properties of polythiophene derivatives with 1,3,5-triazineunits. Electrochim. Acta.

[15] Data P., Zassowski P., Lapkowski M., Domagala W., Krompiec S., Flak T., Penkala M., Swist A., Soloducho J., Danikiewicz W. (2014). Electrochemical and spectroelectrochemical comparison of alternated monomers and their copolymers based on carbazole and thiophene derivatives. Electrochim. Acta.

[16] Lee S.H., Nakamura T., Tsutsui T. (2001). Synthesis and Characterization of Oligo(9,9-dihexyl-2,7-fluorene ethynylene)s: For Application as Blue Light-Emitting Diode. Org. Lett..

[17] Rodrigues P.C., Berlim L.S., Azevedo D., Saavedra N.C., Prasad P.N., Schreiner W.H., Atvars T.D.Z., Akcelrud L. (2012). Electronic structure and optical properties of an alternated fluorene-benzothiadiazole copolymer: Interplay between experimental and theoretical data. J. Phys. Chem. A.

[18] Kim J., Chae S., Yi A., Hong S., Kim H.J., Suh H. (2018). Characterization of push-pull type of conjugated polymers containing 8H-thieno [2,3-b]indole for organic photovoltaics. Synth. Met..

[19] Herguth P., Jiang X., Liu M.S., Jen A.K.Y. (2002). Highly efficient fluorene- and benzothiadiazole-based conjugated copolymers for polymer light-emitting diodes. Macromolecules.

[20] Bujak P., Kulszewicz-Bajer I., Zagorska M., Maurel V., Wielgus I., Pron A. (2013). Polymers for electronics and spintronics. Chem. Soc. Rev..

[21] Hai J., Zhu E., Bian L., Wang J., Wang Z., Li Y., Yin L., Zhang F., Tang W. (2013). Synthesis, optical, electrochemical and electroluminescent properties of novel fluorene-Alt-bithiophene copolymers bearing phenylvinyl bridged accepting side chains. Eur. Polym. J..

[22] Xie L.H., Yin C.R., Lai W.Y., Fan Q.L., Huang W. (2012). Polyfluorene-based semiconductors combined with various periodic table elements for organic electronics. Prog. Polym. Sci..

[23] Güneş A., Cihaner A., Önal A.M. (2013). Synthesis and electro-optical properties of new conjugated hybrid polymers based on furan and fluorene units. Electrochim. Acta.

[24] de Morais A., Duarte L.G.T.A., Turchetti D.A., Mendes R.A., de Freitas J.N., Atvars T.D.Z., Cristovan F.H., Domingues R.A. (2022). Synthesis and optical properties of a fluorene-benzothiadiazole anthracene copolymer. Synth. Met..

[25] Frisch M.J., Trucks G.W., Schlegel H.B., Scuseria G.E., Robb M.A., Cheeseman J.R., Scalmani G., Barone V., Petersson G.A., Nakatsuji H. (2016). Gaussian 16, Rev. C.01. Gaussian 16, Rev. C. 01.

[26] Lane P.A. (2004). Polyfluorene Electroluminescence. Organic Light-Emitting Devices.

[27] Gudeika D., Bezvikonnyi O., Volyniuk D., Grazulevicius J.V. (2020). Differently substituted benzonitriles for non-doped OLEDs. Dye. Pigment..

[28] Masui K., Nakanotani H., Adachi C. (2013). Analysis of exciton annihilation in high-efficiency sky-blue organic light-emitting diodes with thermally activated delayed fluorescence. Org. Electron..

[29] Mennucci B. (2012). Polarizable continuum model. WIREs Comput. Mol. Sci..

