# Supplementary material for: The Effect of Molecular Structure on the Properties of Fluorene Derivatives for OLED Applications
Source: Molecules. 2024 Oct 17;29(20):4918. doi: 10.3390/molecules29204918 (PMC11510416; doi:10.3390/molecules29204918)
Supplement: Supplementary file 1 [file molecules-29-04918-s001.zip › molecules-3225544-supplementary.pdf]

SUPPLEMENTARY INFORMATION

The effect of molecular structure on the properties of fluorene derivatives for OLED applications

Anna Pidluzhna[*,a], Aivars Vembris[a], Raitis Grzibovskis[a], Margarita Anna Zommere[a], Oleksandr Bezvikonnyi[*,b-d], Jurate Simokaitiene[b], Melita Baronaite[b], Dmytro Volyniuk[b], Juozas V. Grazulevicius[b], Amjad Ali[e], Glib Baryshnikov[e,f], Khrystyna Ivaniuk[g], Hryhorii Starykov[b], Pavlo Stakhira[g]

[a] *Institute of Solid State Physics, University of Latvia, Kengaraga str. 8, Riga LV-1063 Latvia. *Corresponding author: anna.pidluzhna@cfi.lu.lv*

[b] *Department of Polymer Chemistry and Technology, Faculty of Chemical Technology, Kaunas University of Technology, K. Baršausko st. 59, LT-51423 Kaunas, Lithuania *Corresponding author: o.bezvikonnyi@ktu.lt*

[c] *Department of Physics, Faculty of Mathematics and Natural Sciences, Kaunas University of Technology, Studentų st. 50, LT-51369 Kaunas, Lithuania*

[d] *KTU "M-Lab" laboratary center, Kaunas University of Technology, Studentų st. 63A, LT-51369 Kaunas, Lithuania*

[e] *Laboratory of Organic Electronics, Department of Science and Technology, Linköping University, Norrköping, SE-60174, Sweden*

[f] *Department of Chemistry and Nanomaterials Science, Bohdan Khmelnytsky National University, Cherkasy, 18031, Ukraine*

[g] *Department of Electronic Engineering, Institute of Telecommunications, Radioelectronics and Electronic Engineering, Lviv Polytechnic National University, Stepan Bandera 12, 79013, Lviv, Ukraine*

Table S1. Emission decay characteristics of the solid samples compounds **1-4**

Sample	λ^{em}_{max}, nm	τ_1, ns	B, %	τ_2, ns	B, %	λ^{em}_{max2}, nm	τ_1, ns	B, %	τ_2, ns	B, %
**1**	396	0.7005	94	1.536	6	416	0.7821	85	1.6060	15
**2**	386	0.7850	100	-	-	406	0.8139	94	1.6530	6
**3**	400	1.0000	100	-	-	419	1.0370	100	-	-
**4**	399	0.7625	100	-	-	419	0.8301	98	2.5940	2

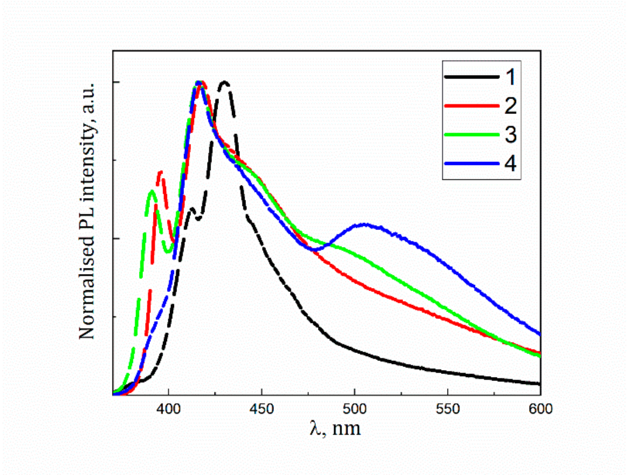

Figure S1. Photoluminescence intensity of 1-4 compounds in thin film state without encapsulation

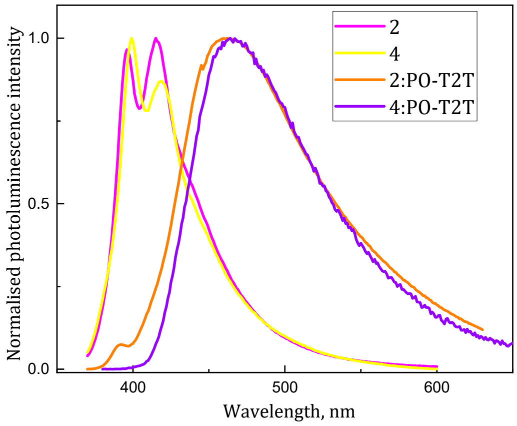

Figure S2. Photoluminescence intensity of 2 and 4 compounds in thin film state encapsulated (magenta and yellow plots) and in mixture with PO-T2T (orange and violet plots)

Table S2. EL characteristics of the fabricated devices

Device, Emitting layers	Von, V	V^{Bright}, V*	Brightness (Cd*m^{-2})	EQE (%)	U_C, V	Max Current efficiency (Cd*A^{-1})	U_P, V	Max Power efficiency (lm*W^{-1})	x	y
**A 4/PO-T2T**	3.8	7.8	520.3	0.18	5.4	0.80	4.6	0.51	0.2406	0.3510
**B 4/1**	3.7	10.2	37.1	0.10	3.6	0.93	3.7	0.81	0.2341	0.2890
**C 2/1**	4.7	9.0	14.9	0.14	4.7	0.52	4.8	0.33	0.1896	0.1544
**D 2/PO-T2T**	4.1	5.8	27.3	0.24	4.8	0.65	4.6	0.43	0.2218	0.3183
**E 4:PO-T2T thick**	3.9	8.8	1182.1	0.42	8.4	1.40	5.0	0.73	0.1992	0.2603
**F 4:PO-T2T thin**	3.4	6.6	1479.2	0.45	4.6	1.19	4.4	0.83	0.1623	0.1942

Von, V is turn on voltage of OLED; **V^{Bright}, V** – Voltage for maximal brightness; **Brightness, (cd*m^{-2})** - Maximal value for Brightness; **EQE (%)** is the External Quantum Efficiency at maximal brightness; **U_C, V** is the voltage at maximal current efficiency; **Max Current efficiency (cd*A^{-1})** is the maximal current efficiency; **U_P, V** is the voltage at maximal power efficiency; **Max Power efficiency (lm*W^{-1})** is the maximal power efficiency; **x** and **y** are CIE 1931 coordinates.

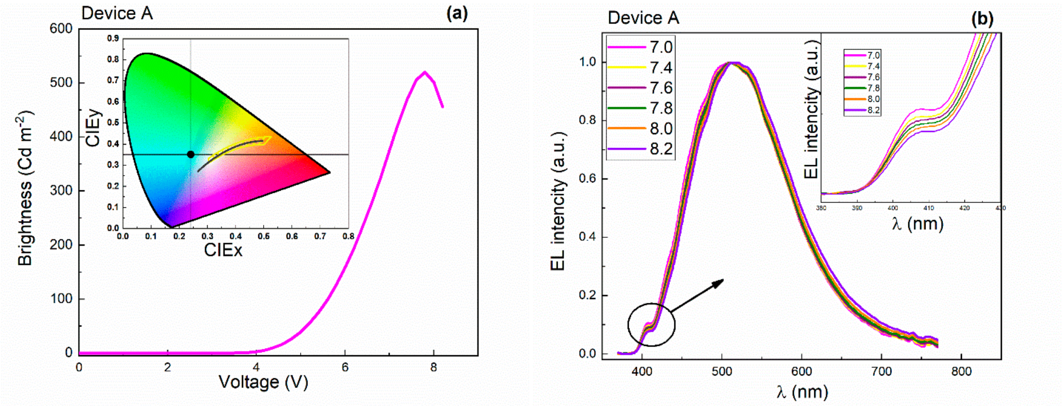

Figure S3. The brightness and CIE1931 chromaticity diagram (a) and EL spectra of the Device A at the different applied voltages (b)

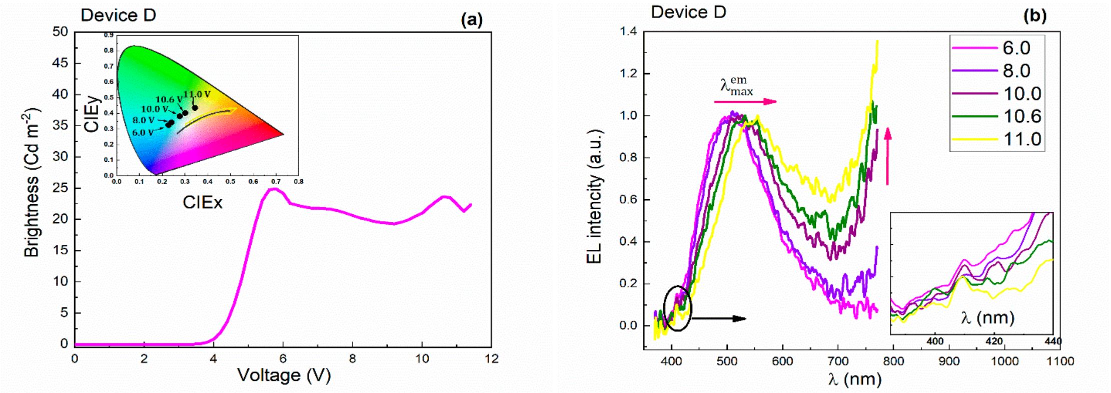

Figure S4. The brightness and CIE1931 chromaticity diagram (a) and EL spectra of the Device D at the different applied voltages (b)

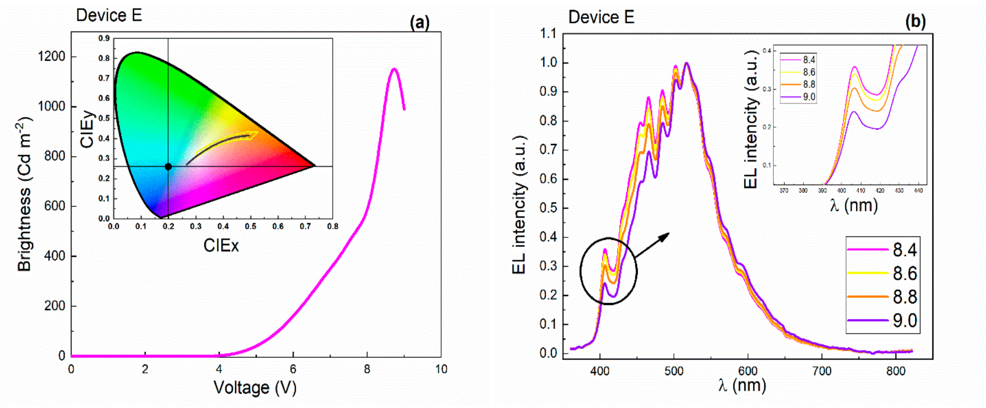

Figure S5. The brightness and CIE1931 chromaticity diagram (a) and EL spectra of the Device E at the different applied voltages (b)

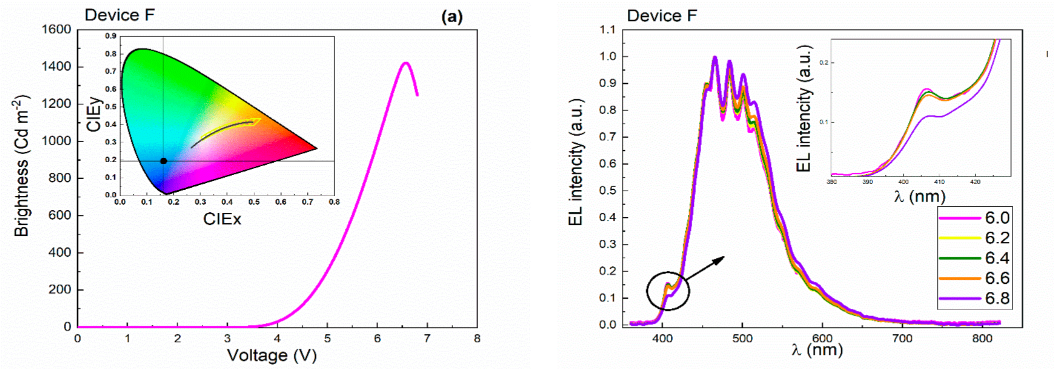

Figure S6. The brightness and CIE1931 chromaticity diagram (a) and EL spectra of the Device F at the different applied voltages (b)

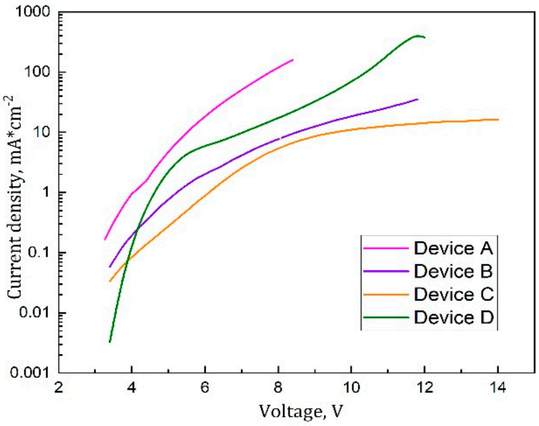

Figure S7. Current density – voltage plots for Devices A-D

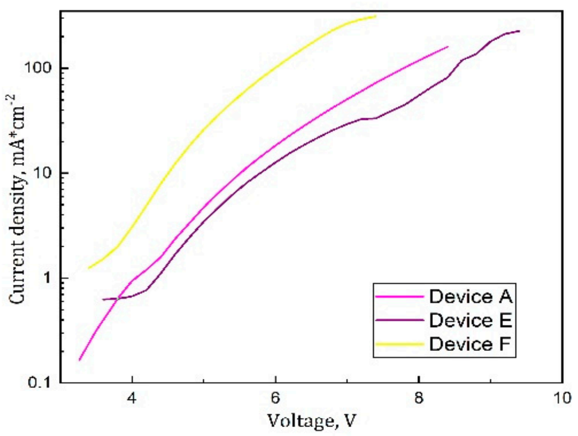

Figure S8. Current density – voltage plots for Devices A, E, F

Below are the cartesian coordinates for all the studied set of molecules optimized in THF solvent using DFT-B3LYP/6-31G(d,p) for ground state, DFT-CAM-B3LYP/6-31G(d,p) for the first singlet excited state, and PCM solvent model. All data is in Å.

Molecule 1

S0				S1			
C	3.46703200	-2.01667800	0.04994600	C	3.45262500	1.98140000	-0.04402000
C	2.53848300	-0.95018100	0.04116600	C	2.51570400	0.90082900	-0.03918300
C	1.18124100	-1.22401700	-0.00482100	C	1.17776800	1.16318700	0.00651800
C	0.72988600	-2.56235000	-0.00230200	C	0.70318400	2.51617700	0.00752200
C	1.63782800	-3.62465400	0.00954400	C	1.62690500	3.59474000	0.00675700
C	3.00070000	-3.34942900	0.02863600	C	2.96667500	3.32595300	-0.01474100
C	0.00000900	-0.25084900	-0.00004200	C	-0.00001500	0.19106600	-0.00002300
C	-1.18125600	-1.22398200	0.00476700	C	-1.17775700	1.16323600	-0.00655300
C	-0.72993800	-2.56232900	0.00221600	C	-0.70311800	2.51620600	-0.00753400
C	-2.53849300	-0.95011500	-0.04120100	C	-2.51570500	0.90093200	0.03914100
C	-3.46706700	-2.01658900	-0.05000500	C	-3.45258100	1.98154200	0.04399500
C	-3.00076900	-3.34935300	-0.02874500	C	-2.96657600	3.32607500	0.01473900
C	-1.63790500	-3.62461100	-0.00966400	C	-1.62679400	3.59480700	-0.00675200
C	0.07479100	0.60858000	1.29310800	C	0.07560500	-0.66401700	-1.28826800
C	-0.07474600	0.60846900	-1.29328300	C	-0.07566900	-0.66402400	1.28821500
C	-1.02148500	1.65757400	1.49327500	C	-1.01173200	-1.71588500	-1.48859600
C	-0.78231600	2.50534500	2.74697800	C	-0.77298100	-2.54992500	-2.74514800
C	-1.87233900	3.55486300	2.98084900	C	-1.85287600	-3.60215000	-2.97983200
C	-1.63745100	4.40686900	4.23187200	C	-1.61835100	-4.44035800	-4.23390400
C	-2.73184800	5.45307300	4.45699200	C	-2.70303000	-5.48921500	-4.45932700
C	1.02153600	1.65744200	-1.49351400	C	1.01162100	-1.71594400	1.48853100
C	0.78246000	2.50503500	-2.74735400	C	0.77284600	-2.54997200	2.74508600
C	1.87247000	3.55455800	-2.98126000	C	1.85268900	-3.60225400	2.97975200
C	1.63768100	4.40638300	-4.23242500	C	1.61814200	-4.44044800	4.23382800
C	2.73206300	5.45259500	-4.45757800	C	2.70276900	-5.48936300	4.45923200
C	4.86149500	-1.74495600	0.08501000	C	4.81929900	1.72971700	-0.07956800
C	-4.86152200	-1.74482600	-0.08504400	C	-4.81926600	1.72991500	0.07953800
C	6.05370200	-1.50138100	0.11456800	C	6.02194100	1.49735100	-0.11074200
C	-6.05372900	-1.50123900	-0.11456400	C	-6.02191300	1.49758000	0.11071200
C	7.44617100	-1.21909700	0.14904400	C	7.39839600	1.23284600	-0.14605500
C	-7.44620800	-1.21899600	-0.14895800	C	-7.39835800	1.23301800	0.14604900
C	7.91182200	0.11101200	0.16248200	C	7.88209700	-0.09416700	-0.17482100
C	9.27317400	0.38189800	0.19705100	C	9.23743700	-0.34698800	-0.20871700
C	10.22282700	-0.65477500	0.21856300	C	10.17683000	0.69721000	-0.21796900
C	9.75285800	-1.97989300	0.20413600	C	9.69326700	2.01566300	-0.19006700
C	8.39377400	-2.26202000	0.17071000	C	8.34115200	2.28487700	-0.15400600
C	-8.39377600	-2.26194600	-0.17083700	C	-8.34115500	2.28501200	0.15403000
C	-9.75286800	-1.97985700	-0.20418800	C	-9.69325900	2.01574300	0.19011500
C	-10.22288100	-0.65475000	-0.21832000	C	-10.17676800	0.69727100	0.21801200
C	-9.27326100	0.38195100	-0.19658900	C	-9.23733400	-0.34688900	0.20872900
C	-7.91190200	0.11110100	-0.16209800	C	-7.88200400	-0.09401400	0.17480900
C	11.67324800	-0.36035200	0.25558700	C	11.62516300	0.41828100	-0.25574900
C	-11.67331100	-0.36036700	-0.25526800	C	-11.62508900	0.41828400	0.25581900
C	12.55733500	-1.18296600	0.97418100	C	12.50666700	1.27850100	-0.92343000
C	13.92281300	-0.90617300	1.00898400	C	13.86937100	1.01611600	-0.95867600

C	14.43396100	0.19976100	0.32645900	C	14.38305100	-0.11246300	-0.32721400
C	13.56676200	1.02603900	-0.39145100	C	13.51966100	-0.97610700	0.33979900
C	12.20135600	0.74883800	-0.42666000	C	12.15690500	-0.71397500	0.37517500
C	-12.20146500	0.74860100	0.42730800	C	-12.15679900	-0.71398500	-0.37510800
C	-13.56687900	1.02576100	0.39217100	C	-13.51954400	-0.97617200	-0.33970600
C	-14.43404000	0.19966800	-0.32599700	C	-14.38295500	-0.11257100	0.32733700
C	-13.92284800	-0.90604400	-1.00884900	C	-13.86930700	1.01602000	0.95880200
C	-12.55736300	-1.18280000	-0.97411600	C	-12.50661400	1.27846000	0.92353000
H	2.91166100	0.06679700	0.08082400	H	2.89910500	-0.11183600	-0.08203300
H	1.29000600	-4.65364400	0.01022200	H	1.27187300	4.62006500	0.01746200
H	3.72310000	-4.15904400	0.03680500	H	3.69029000	4.13392500	-0.01690700
H	-2.91165000	0.06687200	-0.08081500	H	-2.89914700	-0.11171800	0.08197400
H	-3.72319000	-4.15894900	-0.03694000	H	-3.69015700	4.13407600	0.01691800
H	-1.29010500	-4.65360900	-0.01036500	H	-1.27172000	4.62011700	-0.01744100
H	1.04936800	1.11207900	1.30495100	H	1.05233400	-1.16146800	-1.30333000
H	0.07933600	-0.07627900	2.15056800	H	0.07546900	0.01966800	-2.14560000
H	-0.07926400	-0.07647100	-2.15067800	H	-0.07549800	0.01965500	2.14555300
H	-1.04932600	1.11196100	-1.30519500	H	-1.05242000	-1.16143000	1.30327800
H	-1.99824400	1.16709600	1.57752500	H	-1.99156700	-1.23270700	-1.56483700
H	-1.07815600	2.32078500	0.62033600	H	-1.05938000	-2.38538700	-0.62106800
H	0.19382200	3.00554200	2.66644900	H	0.20612700	-3.04293300	-2.67344800
H	-0.71664600	1.84623600	3.62468200	H	-0.71680800	-1.88497500	-3.61756200
H	-2.84819000	3.05376100	3.06115100	H	-2.83173000	-3.10844300	-3.05165700
H	-1.93876500	4.21232200	2.10137300	H	-1.90993700	-4.26563000	-2.10582800
H	-0.66178400	4.90630200	4.15055000	H	-0.63985500	-4.93242000	-4.16110900
H	-1.57132200	3.74859100	5.10943800	H	-1.56157400	-3.77622300	-5.10594100
H	-2.54043800	6.04797500	5.35723500	H	-2.51307600	-6.07621800	-5.36324800
H	-3.71335500	4.97705100	4.57250000	H	-3.68722400	-5.01995200	-4.56617800
H	-2.79722800	6.14339200	3.60696000	H	-2.75887800	-6.18474000	-3.61466400
H	1.07813100	2.32077800	-0.62066400	H	1.05922500	-2.38544900	0.62100100
H	1.99830500	1.16695900	-1.57761300	H	1.99147900	-1.23281300	1.56475800
H	0.71689900	1.84580500	-3.62497600	H	0.71672200	-1.88501900	3.61750100
H	-0.19370400	3.00520700	-2.66698800	H	-0.20628900	-3.04292900	2.67340100
H	1.93878300	4.21214300	-2.10186800	H	1.90970100	-4.26573700	2.10574700
H	2.84834600	3.05347900	-3.06139100	H	2.83157100	-3.10859800	3.05155900
H	1.57166500	3.74798000	-5.10990600	H	1.56141600	-3.77631000	5.10586500
H	0.66198700	4.90579200	-4.15127300	H	0.63961900	-4.93245900	4.16105000
H	2.54072100	6.04736700	-5.35792200	H	2.51280000	-6.07635500	5.36315700
H	2.79733500	6.14303100	-3.60763400	H	2.75856500	-6.18489100	3.61456900
H	3.71359800	4.97659100	-4.57292400	H	3.68699000	-5.02015200	4.56606600
H	7.19355600	0.92456100	0.15527400	H	7.17268800	-0.91458600	-0.17962500
H	9.60740700	1.41406600	0.23322700	H	9.58137500	-1.37482100	-0.25604100
H	10.46459100	-2.79947000	0.19411500	H	10.39633700	2.84163200	-0.16969800
H	8.05033700	-3.29131100	0.15132200	H	7.98851100	3.31001400	-0.12166400
H	-8.05030100	-3.29122800	-0.15167200	H	-7.98855500	3.31016300	0.12169300
H	-10.46457800	-2.79945700	-0.19435300	H	-10.39636100	2.84168400	0.16976900
H	-9.60753400	1.41411400	-0.23251800	H	-9.58123100	-1.37473700	0.25604800
H	-7.19365800	0.92466900	-0.15471500	H	-7.17256300	-0.91440500	0.17959000
H	12.16737200	-2.03018700	1.53026600	H	12.11665500	2.14762900	-1.44305000
H	14.58728600	-1.55073600	1.57741800	H	14.53234300	1.69233300	-1.48930700
H	15.49804300	0.41561800	0.35374500	H	15.44844200	-0.31744500	-0.35480200
H	13.95485900	1.88415000	-0.93286200	H	13.91011500	-1.85507500	0.84312300
H	11.54009200	1.38289300	-1.00957700	H	11.49881100	-1.38146900	0.92183000
H	-11.54022200	1.38250600	1.01041300	H	-11.49869100	-1.38144600	-0.92178500
H	-13.95501700	1.88369300	0.93383600	H	-13.90997400	-1.85514900	-0.84303300
H	-15.49813000	0.41549600	-0.35323000	H	-15.44833600	-0.31759700	0.35494600
H	-14.58729700	-1.55045700	-1.57748300	H	-14.53229300	1.69220400	1.48945700
H	-12.16736200	-2.02985000	-1.53043600	H	-12.11662500	2.14759700	1.44315300

Molecule 2

S0				S1			
C	3.50072500	-1.92009300	0.10333700	C	3.48862700	-1.89703900	0.09964300
C	2.54654000	-0.87631500	0.08595900	C	2.52349400	-0.83959900	0.08485800
C	1.19714100	-1.18143400	0.01119300	C	1.19417700	-1.13361700	0.01087500
C	0.77657700	-2.52960200	-0.00693400	C	0.74995800	-2.49904800	-0.01108700
C	1.70956500	-3.56972900	0.01401000	C	1.70289600	-3.55472200	-0.00018500
C	3.06511300	-3.26283000	0.06186500	C	3.03441700	-3.25269800	0.05063400
C	-0.00655700	-0.23585300	0.00120000	C	-0.00722700	-0.18988600	0.00249600
C	-1.16514700	-1.23587100	-0.02726700	C	-1.16203600	-1.18980000	-0.02399600
C	-0.68273000	-2.56326900	-0.03219400	C	-0.65310400	-2.53260400	-0.02469000
C	-2.52707600	-0.99224500	-0.09910900	C	-2.50376500	-0.95853600	-0.09543300
C	-3.43205100	-2.07847000	-0.13642000	C	-3.41730400	-2.06045800	-0.13009800
C	-2.93496900	-3.40016900	-0.11718600	C	-2.89896400	-3.39353500	-0.10273100
C	-1.56667000	-3.64491400	-0.07245500	C	-1.55453400	-3.63234900	-0.05462300
C	0.02193100	0.61196500	1.30381100	C	0.02168000	0.65432100	1.29970100
C	-0.07541900	0.63480400	-1.28460400	C	-0.07766700	0.67657500	-1.27822800
C	-1.10147500	1.63440400	1.49047700	C	-1.09336000	1.67932300	1.48666900
C	-0.91063800	2.47297400	2.75859600	C	-0.90413400	2.50337700	2.75815200
C	-2.02930400	3.49519200	2.97757000	C	-2.01312800	3.52810200	2.97839200

C	-1.84415500	4.33809400	4.24305500	C	-1.82903700	4.35618700	4.24753500
C	-2.96740500	5.35655700	4.45272900	C	-2.94291400	5.37717900	4.45805900
C	1.00077500	1.70981500	-1.45336700	C	0.98778600	1.75604900	-1.44706600
C	0.76548500	2.56683000	-2.70150100	C	0.75390700	2.59747500	-2.69959000
C	1.83667700	3.64189500	-2.90433100	C	1.81297400	3.67705900	-2.90292300
C	1.60536700	4.50345100	-4.14947200	C	1.58285400	4.52280900	-4.15274500
C	2.68103200	5.57500300	-4.34353800	C	2.64672000	5.59893500	-4.34670400
C	4.88789500	-1.61681000	0.16625300	C	4.84850400	-1.61295400	0.16251400
C	-4.83148200	-1.83753900	-0.19744800	C	-4.78884500	-1.83939100	-0.19183600
C	6.07438600	-1.34932500	0.21864800	C	6.04481600	-1.35423400	0.21682500
C	-6.02804100	-1.61914800	-0.24872300	C	-5.99528300	-1.63249100	-0.24565400
C	7.45979900	-1.03727900	0.27900900	C	7.41615500	-1.05898700	0.27726800
C	-7.42610700	-1.36959800	-0.30801700	C	-7.37831400	-1.39789100	-0.30601800
C	7.89861400	0.30498700	0.31337300	C	7.87110300	0.28021400	0.32132400
C	9.24833600	0.60828300	0.37184400	C	9.21566800	0.56607400	0.37986200
C	10.20574300	-0.41938300	0.39803200	C	10.16138800	-0.46953000	0.39698900
C	9.78889000	-1.75706700	0.36430200	C	9.73397800	-1.80046400	0.35425100
C	8.42989800	-2.05501700	0.30562400	C	8.38074100	-2.08557200	0.29543300
C	-8.35091200	-2.43622200	-0.35722900	C	-8.29876800	-2.47186000	-0.34751200
C	-9.71249700	-2.19159800	-0.41474200	C	-9.65315300	-2.23707900	-0.40634500
C	-10.19662900	-0.87292200	-0.42523500	C	-10.14584400	-0.92402600	-0.42626500
C	-9.29384700	0.19803800	-0.37683000	C	-9.25404800	0.15252100	-0.38596900
C	-7.92573000	-0.05510200	-0.31904200	C	-7.89186700	-0.08619100	-0.32694200
O	11.50345500	-0.01721200	0.45614300	O	11.45539200	-0.08317700	0.45550300
O	-11.54872000	-0.74013100	-0.48351900	O	-11.49071700	-0.80162900	-0.48475200
C	-12.09348100	0.57651000	-0.49833400	C	-12.04786000	0.50210700	-0.50768100
C	12.51565000	-1.01975400	0.48632400	C	12.45729500	-1.08641100	0.47549200
H	2.89430300	0.14898400	0.14143800	H	2.88058200	0.18207100	0.14322400
H	1.38662400	-4.60676000	-0.00068500	H	1.37435700	-4.58868300	-0.02623700
H	3.80569900	-4.05585100	0.07710800	H	3.77724400	-4.04327900	0.06094700
H	-2.92175700	0.01665600	-0.13783000	H	-2.90925000	0.04569700	-0.13749200
H	-3.63810800	-4.22615400	-0.14749100	H	-3.60314000	-4.21842700	-0.12763800
H	-1.19631200	-4.66613500	-0.07538000	H	-1.17707000	-4.64981900	-0.04539600
H	0.98468300	1.13653400	1.34171600	H	0.98651000	1.17324400	1.34099000
H	0.02329400	-0.08136700	2.15447500	H	0.01849200	-0.03780200	2.15028000
H	-0.04800000	-0.04132500	-2.14856800	H	-0.04395000	0.00172100	-2.14191500
H	-1.06067000	1.11661000	-1.31082000	H	-1.06576000	1.15057500	-1.30848900
H	-2.06873800	1.12160700	1.54648100	H	-2.06342600	1.17325300	1.53326500
H	-1.15287700	2.30625400	0.62380500	H	-1.13555700	2.35811200	0.62602800
H	0.05565200	2.99559600	2.70678100	H	0.06496300	3.01914700	2.71637000
H	-0.85092800	1.80560900	3.63047500	H	-0.85405800	1.82941200	3.62402000
H	-2.99518600	2.97144000	3.02870200	H	-2.98189600	3.01152100	3.02006100
H	-2.08953300	4.16097800	2.10391400	H	-2.06392900	4.20075300	2.11100600
H	-0.87854200	4.86043900	4.19087400	H	-0.86069600	4.87134000	4.20485000
H	-1.78407800	3.67159900	5.11483500	H	-1.77838500	3.68298700	5.11297500
H	-2.81145200	5.94553600	5.36365500	H	-2.78894200	5.95745400	5.37311900
H	-3.94019600	4.85705600	4.53927500	H	-3.91830500	4.88424800	4.53499800
H	-3.02820300	6.05472300	3.60878000	H	-2.99409700	6.08140500	3.62032600
H	1.02742300	2.36391100	-0.57213900	H	1.00280500	2.41741800	-0.57208700
H	1.98936300	1.24166100	-1.52532400	H	1.98000100	1.29670000	-1.50849700
H	0.72926900	1.91669700	-3.58759200	H	0.73015000	1.94054300	-3.57957000
H	-0.22242400	3.04526400	-2.63274100	H	-0.23776200	3.06680100	-2.64224800
H	1.87388900	4.29025600	-2.01640800	H	1.83783900	4.33248700	-2.02134300
H	2.82425000	3.16255100	-2.97319200	H	2.80436500	3.20710900	-2.96078300
H	1.56833100	3.85427500	-5.03549600	H	1.55824900	3.86674900	-5.03237300
H	0.61805600	4.98129600	-4.07954200	H	0.59186900	4.99121900	-4.09389900
H	2.49266700	6.17625700	-5.24021200	H	2.46040800	6.19082000	-5.24821800
H	2.71706600	6.25656400	-3.48472600	H	2.67013200	6.28669900	-3.49419200
H	3.67426700	5.12110300	-4.44716600	H	3.64350900	5.15382600	-4.43914800
H	7.16431400	1.10386800	0.29342700	H	7.14455900	1.08536000	0.30822500
H	9.59032200	1.63784500	0.39855700	H	9.57103300	1.59015500	0.41378400
H	10.50824200	-2.56651300	0.38295000	H	10.44718100	-2.61475000	0.36621700
H	8.11065900	-3.09198700	0.27965100	H	8.05175700	-3.11852700	0.26231100
H	-7.98382400	-3.45746800	-0.34956400	H	-7.92385900	-3.48943600	-0.33243700
H	-10.42684000	-3.00761100	-0.45275300	H	-10.36395400	-3.05555100	-0.43846100
H	-9.64312300	1.22322100	-0.38349200	H	-9.61322700	1.17361600	-0.40007800
H	-7.22942900	0.77659400	-0.28157800	H	-7.20221100	0.75032400	-0.29568700
H	-13.17550800	0.45038600	-0.54705800	H	-13.12767500	0.36631300	-0.55509400
H	-11.82963300	1.12617900	0.41294700	H	-11.79001000	1.05946400	0.39909200
H	-11.75052200	1.13867600	-1.37497900	H	-11.71184500	1.06159800	-1.38715000
H	13.46414200	-0.48383900	0.53343000	H	13.40924000	-0.55908400	0.52468900
H	12.41325800	-1.66138400	1.36949600	H	12.35311400	-1.73342100	1.35292100
H	12.48933000	-1.63855600	-0.41842900	H	12.42534700	-1.69694500	-0.43316200

Molecule 3

S0				S1			
C	3.61489300	-1.36980500	0.05055500	C	3.60211300	-1.35233300	0.05520300
C	2.53109800	-0.46139200	0.05481400	C	2.50851400	-0.43213000	0.07085500
C	1.23622300	-0.93992600	-0.05984900	C	1.22962700	-0.89651800	-0.03787300

C	1.00076100	-2.33051900	-0.13945900	C	0.97399700	-2.30255700	-0.12586200
C	2.06358300	-3.23794000	-0.14157800	C	2.05298800	-3.22235200	-0.14511200
C	3.36476500	-2.75575100	-0.05370700	C	3.33397400	-2.75122600	-0.06002800
C	-0.08234100	-0.16321200	-0.06105200	C	-0.08506200	-0.11991800	-0.02906000
C	-1.09522000	-1.30688900	-0.14595200	C	-1.09570100	-1.26144800	-0.10991700
C	-0.44042500	-2.55745100	-0.19323300	C	-0.41825000	-2.52133800	-0.16333900
C	-2.47614800	-1.24450700	-0.22842700	C	-2.45724100	-1.20625100	-0.18384500
C	-3.22668200	-2.43972500	-0.31728400	C	-3.21434500	-2.41508300	-0.27350100
C	-2.55933300	-3.68362100	-0.34360200	C	-2.52702800	-3.66693900	-0.30449600
C	-1.17097300	-3.74513200	-0.28890900	C	-1.16134100	-3.72573100	-0.25434700
C	-0.18991600	0.63603900	1.26790000	C	-0.18306600	0.67326700	1.29661900
C	-0.24627700	0.73351100	-1.32062900	C	-0.25521000	0.77441300	-1.28156200
C	-1.45006500	1.48217900	1.46536600	C	-1.43142700	1.52691200	1.50223800
C	-1.39767500	2.30589100	2.75610900	C	-1.36554500	2.33761700	2.79439500
C	-2.66140100	3.13970800	2.98491300	C	-2.61451300	3.18131700	3.03279400
C	-2.62005100	3.96873500	4.27212000	C	-2.55754000	3.99829800	4.32097600
C	-3.88947100	4.79551900	4.49108700	C	-3.81222700	4.83564500	4.54915600
C	0.67389200	1.95162900	-1.42928900	C	0.65698800	1.99264200	-1.39371400
C	0.35224600	2.80632200	-2.65962600	C	0.33189100	2.83730000	-2.62352800
C	1.26508200	4.02771400	-2.79853200	C	1.23644700	4.05775600	-2.76733000
C	0.94746400	4.88797300	-4.02539700	C	0.91534700	4.90792800	-3.99387000
C	1.86424900	6.10689600	-4.15448200	C	1.82457500	6.12565900	-4.12756000
C	4.94747900	-0.88829100	0.15451500	C	4.91106900	-0.89506400	0.15236100
C	-4.64462200	-2.37532600	-0.37640000	C	-4.60253900	-2.36586400	-0.32762200
C	6.08450200	-0.46225200	0.24261300	C	6.06169700	-0.48088600	0.23716000
C	-5.85835500	-2.29162100	-0.41742200	C	-5.82535300	-2.29450800	-0.36840300
C	7.41484900	0.02571800	0.34427700	C	7.37567400	-0.00911700	0.33259900
C	-7.27306000	-2.16991100	-0.45822900	C	-7.22085400	-2.19746200	-0.41048900
C	8.52538900	-0.88969500	0.36262800	C	8.49105600	-0.92611700	0.32316200
C	9.85162200	-0.36079100	0.46547100	C	9.80929400	-0.40071600	0.42131400
C	10.03998400	1.04418300	0.54634600	C	10.00041500	0.99825600	0.52522000
C	8.96113000	1.90069500	0.52709500	C	8.92300300	1.85741800	0.53307100
C	7.64979700	1.39447300	0.42631300	C	7.61948200	1.36366000	0.43800900
C	-8.06761500	-3.29290200	-0.66396500	C	-8.00341500	-3.34995400	-0.52542300
C	-9.47250200	-3.18917400	-0.70703500	C	-9.39781100	-3.27066200	-0.56995800
C	-10.08724800	-1.96699100	-0.54501300	C	-10.03327000	-2.04994300	-0.50045700
C	-9.31942700	-0.79189100	-0.33116000	C	-9.28796800	-0.85207200	-0.38156200
C	-7.89157000	-0.88152300	-0.28474400	C	-7.86747800	-0.90837700	-0.33448100
C	8.35192000	-2.29426300	0.28274000	C	8.31781000	-2.32176800	0.22044800
C	9.43864000	-3.14091500	0.30333800	C	9.40074200	-3.16837000	0.21447000
C	10.75121200	-2.62086100	0.40493900	C	10.70885400	-2.65182600	0.31156900
C	10.95038500	-1.26129100	0.48402700	C	10.90638700	-1.29878900	0.41258800
C	-9.93365300	0.47769700	-0.15954900	C	-9.92582000	0.41196000	-0.30622100
C	-9.17542500	1.60721900	0.04950800	C	-9.19352400	1.56510800	-0.18865400
C	-7.76367900	1.51538400	0.09611200	C	-7.78580200	1.50485900	-0.14104700
C	-7.13750400	0.29911700	-0.06749500	C	-7.13994600	0.29365900	-0.21278500
H	2.73788000	0.59769600	0.15801300	H	2.72618400	0.62395700	0.17896300
H	1.88214400	-4.30694400	-0.20495300	H	1.86378400	-4.28817600	-0.22063300
H	4.20474500	-3.44279200	-0.05522900	H	4.17435900	-3.43699000	-0.07195000
H	-3.00229900	-0.29685000	-0.23555000	H	-2.99218600	-0.26380900	-0.18607500
H	-3.14578100	-4.59386100	-0.41371400	H	-3.11487700	-4.57581400	-0.37340800
H	-0.66703600	-4.70668500	-0.32428200	H	-0.65163100	-4.68310700	-0.28822400
H	0.68843600	1.28941900	1.33863100	H	0.70008900	1.31877000	1.36733700
H	-0.10181600	-0.07785800	2.09663500	H	-0.09746700	-0.04175300	2.12353700
H	-0.10868200	0.09759400	-2.20433400	H	-0.11914000	0.14159900	-2.16670300
H	-1.28795300	1.07558900	-1.35560900	H	-1.29757500	1.11205300	-1.31364400
H	-2.33371800	0.83431300	1.49589700	H	-2.32075800	0.88860500	1.53121100
H	-1.58974900	2.16224400	0.61498200	H	-1.56700900	2.21432900	0.65829400
H	-0.52161900	2.97014900	2.72916400	H	-0.48306800	2.99138500	2.76952800
H	-1.24521300	1.63259400	3.61195400	H	-1.21799200	1.65718000	3.64387000
H	-3.53609400	2.47323100	3.01088900	H	-3.49583200	2.52575400	3.05824000
H	-2.81472400	3.81027600	2.12638700	H	-2.76352300	3.85863800	2.18046400
H	-1.74653200	4.63518000	4.24459300	H	-1.67730700	4.65352900	4.29406100
H	-2.46549000	3.29769700	5.12863000	H	-2.40754500	3.32067200	5.17129500
H	-3.83495500	5.37853200	5.41740900	H	-3.74795700	5.41160800	5.47751200
H	-4.77372500	4.14937700	4.55350500	H	-4.70263300	4.20033000	4.61087400
H	-4.05016800	5.49676200	3.66293000	H	-3.96764800	5.54297600	3.72702900
H	0.58744700	2.57544200	-0.53014300	H	0.56752500	2.61962900	-0.49816500
H	1.71961100	1.62750800	-1.48592300	H	1.70335300	1.67411100	-1.44915400
H	0.43225700	2.18648000	-3.56430500	H	0.41434600	2.21457900	-3.52452700
H	-0.69502400	3.13806900	-2.60781800	H	-0.71563100	3.16444300	-2.57323000
H	1.18584000	4.64567000	-1.89189100	H	1.15500800	4.67877700	-1.86452600
H	2.31220100	3.69497100	-2.84996800	H	2.28379700	3.72978300	-2.81771400
H	1.02770400	4.26946800	-4.93028200	H	0.99754300	4.28644600	-4.89483300
H	-0.09942000	5.21855500	-3.97326400	H	-0.13153000	5.23398300	-3.94274700
H	1.61658000	6.70445200	-5.03910300	H	1.57526300	6.71826600	-5.01321200
H	1.77918200	6.75810200	-3.27581100	H	1.73739000	6.77924300	-3.25260000
H	2.91452800	5.80199500	-4.23952300	H	2.87463600	5.82469100	-4.21146800
H	11.05202600	1.43219700	0.62376000	H	11.01283600	1.38384000	0.59868700

H	9.11340100	2.97393400	0.58936600	H	9.08210200	2.92778000	0.61330200
H	6.80565800	2.07621400	0.41160500	H	6.77671000	2.04609800	0.44457200
H	-7.59165500	-4.25949500	-0.79309200	H	-7.51079100	-4.31442800	-0.58120000
H	-10.06684700	-4.08315500	-0.86966900	H	-9.97998200	-4.18187500	-0.66040900
H	-11.17027900	-1.88474100	-0.57797500	H	-11.11692900	-1.99029900	-0.53539100
H	7.34469000	-2.69085200	0.20496300	H	7.31096200	-2.71868000	0.14599800
H	9.28937500	-4.21492400	0.24136300	H	9.24911300	-4.23997900	0.13478300
H	11.59962900	-3.29868500	0.42020200	H	11.55761800	-3.32809500	0.30613000
H	11.95473800	-0.85340700	0.56233000	H	11.91047700	-0.89167100	0.48806500
H	-11.01814200	0.53898800	-0.19629700	H	-11.01070000	0.44826000	-0.34302000
H	-9.65720700	2.57180200	0.17973800	H	-9.69452600	2.52609800	-0.13150100
H	-7.17182000	2.41077300	0.26250000	H	-7.21007600	2.41997400	-0.04692700
H	-6.05527300	0.22676800	-0.03129400	H	-6.05678700	0.24713400	-0.17546900

Molecule 4

S0				S1			
C	-3.44252300	-2.32989400	-0.06864800	C	-3.43125000	-2.30642200	-0.06239100
C	-2.53033500	-1.24922600	-0.05213800	C	-2.50912400	-1.21140800	-0.04830800
C	-1.16905000	-1.50176300	-0.00491300	C	-1.16770800	-1.45361900	-0.00146000
C	-0.69611000	-2.83260900	-0.01424400	C	-0.67146900	-2.79828800	-0.00803200
C	-1.58746300	-3.90880900	-0.03381200	C	-1.57848100	-3.89052200	-0.01827000
C	-2.95455900	-3.65505700	-0.05383100	C	-2.92245200	-3.64346000	-0.04112300
C	-0.00340400	-0.50976600	-0.00162500	C	-0.00488600	-0.46302300	0.00059700
C	1.19352700	-1.46370800	-0.00998200	C	1.18851400	-1.41620600	-0.00986100
C	0.76349100	-2.80904600	-0.01626000	C	0.73605300	-2.77540700	-0.01980600
C	2.54611900	-1.16813600	0.03970300	C	2.52278200	-1.13194700	0.03917300
C	3.49239000	-2.21916100	0.04308200	C	3.47730900	-2.19539200	0.03880300
C	3.04708400	-3.55901000	0.01292100	C	3.01379400	-3.54564800	0.00144200
C	1.68886400	-3.85620800	-0.00959100	C	1.67754400	-3.83695300	-0.02352500
C	-0.08965400	0.35598800	-1.28977900	C	-0.09182300	0.39929900	-1.28197500
C	0.05488300	0.34313600	1.29665300	C	0.05469700	0.38498800	1.29426100
C	0.99050900	1.42297600	-1.48240100	C	0.97941700	1.46904700	-1.47432800
C	0.73926800	2.27504300	-2.73080700	C	0.72875400	2.30856500	-2.72488200
C	1.81323900	3.34260300	-2.95726000	C	1.79290900	3.37842000	-2.95169200
C	1.56611200	4.19881700	-4.20303800	C	1.54630600	4.22210700	-4.19975300
C	2.64441700	5.26315300	-4.42074900	C	2.61533300	5.28856500	-4.41727200
C	-1.05802100	1.37388600	1.49985900	C	-1.04862000	1.41937600	1.49824700
C	-0.83733000	2.21642400	2.76046100	C	-0.82812500	2.24697300	2.76235200
C	-1.94440400	3.24749100	2.99632200	C	-1.92430900	3.28163200	2.99965200
C	-1.72890900	4.09426600	4.25437700	C	-1.70866000	4.11333900	4.26141600
C	-2.84057800	5.12172200	4.48123600	C	-2.80977300	5.14442100	4.48920000
C	-4.84088300	-2.07958000	-0.10471500	C	-4.79959700	-2.07553000	-0.09884700
C	4.88241800	-1.92603500	0.08124500	C	4.84196300	-1.92079000	0.07673100
C	-6.03559100	-1.84739400	-0.13490000	C	-6.00638000	-1.85655200	-0.13061200
C	6.07024200	-1.66136400	0.11322100	C	6.03923100	-1.66720700	0.10948600
C	-7.43378600	-1.59490800	-0.17001900	C	-7.38386300	-1.61414300	-0.16611400
C	7.46141500	-1.37196300	0.15014600	C	7.41449900	-1.38807800	0.14604700
C	-8.35765500	-2.68853400	-0.19381700	C	-8.30939100	-2.71090700	-0.18331000
C	-9.70942200	-2.46508900	-0.22789300	C	-9.65074100	-2.49242100	-0.21786600
C	-10.23701100	-1.14167600	-0.24090000	C	-10.18399200	-1.16813100	-0.23824800
C	-9.31718500	-0.04089600	-0.21706200	C	-9.27127400	-0.06902500	-0.22122500
C	-7.92646900	-0.29609400	-0.18186900	C	-7.88880100	-0.31287400	-0.18556700
C	7.91985700	-0.05892500	0.18431500	C	7.88407300	-0.07557600	0.18259400
C	9.30096400	0.23285100	0.22141800	C	9.25897600	0.20447900	0.21910900
C	10.24957000	-0.83986300	0.22373000	C	10.19857900	-0.86661700	0.21902500
C	9.76039200	-2.17431400	0.18807300	C	9.70355700	-2.20024800	0.18136200
C	8.41349900	-2.43719000	0.15261100	C	8.36573600	-2.45681200	0.14629100
C	9.79080700	1.56955500	0.25724400	C	9.75729000	1.53865100	0.25685500
C	11.13586000	1.82798600	0.29334100	C	11.09641200	1.78725500	0.29210000
C	12.07486800	0.75836500	0.29574000	C	12.02643800	0.71381400	0.29190500
C	11.63861300	-0.55248600	0.26124900	C	11.58340600	-0.58958700	0.25580500
C	-11.62354700	-0.88482500	-0.27627800	C	-11.56345500	-0.92071700	-0.27405900
C	-12.10508600	0.41382000	-0.28821900	C	-12.05210400	0.37210500	-0.29299500
C	-11.20011700	1.50828500	-0.26445800	C	-11.15686000	1.46917800	-0.27608800
C	-9.84326700	1.27598500	-0.22990700	C	-9.80575700	1.24529100	-0.24127100
O	-13.45959800	0.55698000	-0.32306600	O	-13.39983800	0.50604800	-0.32748100
O	13.37765000	1.14809500	0.33383300	O	13.32546900	1.09146600	0.32911700
C	14.37430200	0.13061400	0.34056000	C	14.31216200	0.07458500	0.33286900
C	-14.00427600	1.87321500	-0.33829300	C	-13.95416400	1.81049400	-0.34915200
H	-2.91932100	-0.23800600	-0.08697400	H	-2.90771100	-0.20432800	-0.08561800
H	-1.22369000	-4.93233800	-0.03962500	H	-1.20763300	-4.91043900	-0.01369800
H	-3.66384900	-4.47615200	-0.06785100	H	-3.63261900	-4.46335800	-0.05039300
H	2.90249700	-0.14537900	0.08600800	H	2.88848200	-0.11290600	0.08827400
H	3.78234500	-4.35704800	0.01699400	H	3.75004700	-4.34228800	0.00008200
H	1.35795200	-4.89075000	-0.01574300	H	1.34051700	-4.86835200	-0.04031600
H	-1.07193500	0.84437200	-1.30020100	H	-1.07613900	0.88172500	-1.29548700
H	-0.08238700	-0.32387400	-2.15119700	H	-0.07995700	-0.27882100	-2.14366500
H	0.06762800	-0.34663200	2.15009700	H	0.06206500	-0.30421300	2.14717000
H	1.02158000	0.86146900	1.31420700	H	1.02396000	0.89670500	1.31568300

H	1.97469800	0.94804700	-1.56892800	H	1.96644000	1.00125200	-1.55336500
H	1.03641600	2.08133100	-0.60515200	H	1.01663800	2.13290200	-0.60191200
H	-0.24446900	2.75975400	-2.64785900	H	-0.25762500	2.78646800	-2.65012000
H	0.68423700	1.62063800	-3.61275500	H	0.68270800	1.64917300	-3.60211300
H	2.79670000	2.85704600	-3.04002100	H	2.77901000	2.89985300	-3.02673500
H	1.86904500	3.99543600	-2.07360100	H	1.83995000	4.03637000	-2.07290300
H	0.58280800	4.68265000	-4.11931500	H	0.56057500	4.69905000	-4.12378600
H	1.51070100	3.54515900	-5.08478500	H	1.49961400	3.56350400	-5.07657100
H	2.44449600	5.86069900	-5.31739000	H	2.41696600	5.87916300	-5.31704600
H	3.63321100	4.80303900	-4.53847400	H	3.60641300	4.83474200	-4.52708700
H	2.69859000	5.94903400	-3.56634300	H	2.66062800	5.97876900	-3.56761800
H	-1.12130800	2.04234000	0.63135800	H	-1.10218600	2.09497600	0.63575000
H	-2.02747200	0.86795300	1.57651200	H	-2.02153800	0.92122700	1.56593300
H	-0.76568600	1.55231600	3.63391200	H	-0.76672800	1.57614300	3.62990700
H	0.13142800	2.73196700	2.68799900	H	0.14411700	2.75461200	2.69957600
H	-2.01666600	3.91002100	2.12110700	H	-1.98650300	3.95108200	2.13053800
H	-2.91280000	2.73089400	3.06825600	H	-2.89624400	2.77321700	3.06241300
H	-1.65687300	3.43097700	5.12768300	H	-1.64671900	3.44330300	5.12856700
H	-0.76074800	4.60935100	4.18142100	H	-0.73711500	4.62021800	4.19768800
H	-2.66289800	5.71328000	5.38648800	H	-2.63320200	5.72709900	5.39862500
H	-2.91257800	5.81683200	3.63564700	H	-2.87144400	5.84562400	3.64963800
H	-3.81507600	4.62965500	4.58857500	H	-3.78750200	4.65994300	4.58719100
H	-7.96596900	-3.70058500	-0.18431000	H	-7.91438500	-3.72093900	-0.16812300
H	-10.40095800	-3.30308200	-0.24571500	H	-10.34127400	-3.33032800	-0.23064400
H	-7.23517600	0.54139900	-0.16391700	H	-7.20103200	0.52674600	-0.17279000
H	7.20567200	0.75935700	0.18269800	H	7.17444100	0.74581800	0.18280600
H	10.47472300	-2.99329300	0.18953800	H	10.41578100	-3.02005200	0.18118100
H	8.05209300	-3.46012200	0.12581600	H	8.00009400	-3.47753300	0.11788500
H	9.07802100	2.38961300	0.25569200	H	9.04895800	2.36159800	0.25712700
H	11.51782200	2.84338900	0.32098200	H	11.48649100	2.79878400	0.32108400
H	12.33782600	-1.38038900	0.26204200	H	12.27756100	-1.42106700	0.25469000
H	-12.33040400	-1.70878500	-0.29480700	H	-12.26743600	-1.74640100	-0.28748700
H	-11.56814700	2.52683800	-0.27325100	H	-11.53087300	2.48489700	-0.29037500
H	-9.15409500	2.11593200	-0.21186600	H	-9.12073000	2.08773600	-0.22835200
H	15.33252700	0.65003300	0.37374700	H	15.27350100	0.58607200	0.36558300
H	14.27487200	-0.51490200	1.22144400	H	14.21102400	-0.57119500	1.21186000
H	14.31967300	-0.48301700	-0.56663000	H	14.25356500	-0.53667700	-0.57421900
H	-15.08695300	1.74485800	-0.36647200	H	-15.03493700	1.67517600	-0.37582400
H	-13.72787900	2.43179900	0.56383300	H	-13.68226800	2.37541300	0.54884300
H	-13.68108500	2.43037700	-1.22562100	H	-13.63683700	2.36501100	-1.23868900
